# Sham tDCS controls: blinding, reliability, and a specification-grade checklist

**DOI:** 10.3389/fnhum.2026.1742370

**Published:** 2026-05-05

**Authors:** Milos Ljubisavljevic, Jonida Basha, Fransina C. King, Fatima Y. Ismail, Shahid Bashir, Yauhen Statsenko, Miklos Szolics, Gordon C. Baylis

**Affiliations:** 1 Department of Physiology, College of Medicine and Health Sciences (CMHS), United Arab Emirates University, Al Ain, United Arab Emirates; 2 Department of Pediatrics, College of Medicine and Health Sciences (CMHS), United Arab Emirates University, Al Ain, United Arab Emirates; 3 Neuroscience Centre, King Fahad Specialist Hospital, Dammam, Saudi Arabia; 4 Department of Radiology, College of Medicine and Health Sciences (CMHS), United Arab Emirates University, Al Ain, United Arab Emirates; 5 Department of Neurology, Sheikh Tahnoon Medical City, United Arab Emirates University, Al Ain, United Arab Emirates; 6 Department of Psychological Sciences, Western Kentucky University, Bowling Green, KY, United States

**Keywords:** blinding, electric-field modeling, non-invasive brain stimulation, reliability, reporting standards, sham control, transcranial direct current stimulation

## Abstract

Sham transcranial direct current stimulation (tDCS) is essential for causal inference but is commonly implemented as ramp-only protocols that may not ensure physiological inertness and may fail to maintain mid-session blinding. We synthesize evidence on sham blinding integrity and test–retest reliability from January 1, 2010, to August 31, 2024, with a targeted post-search update (September 2024–September 2025) for key methodological advances directly informing sham design, blinding assessment, or physiological inertness. Evidence across conventional fade-in/short-stimulation/fade-out (FSF) approaches and emerging HD and device-locked alternatives suggests that end-of-study “guess” measures frequently overestimate blinding, whereas time-resolved probes detect perceptual divergence during stimulation. Test–retest reliability under sham conditions is generally adequate across common behavioral tasks, although practice effects and procedural heterogeneity can reduce stability and inflate variance. Based on recurrent methodological failure factors identified in the mapped literature, we propose five specification-grade recommendations: (i) use time-resolved (not end-of-study) blinding assessment with pre-specified success criteria; (ii) constrain intracranial E-fields below a conservative engineering ceiling using accessible modeling tools; (iii) standardize ramp profiles and electrode preparation and impedance protocols; (iv) implement device-locked randomization with audit trails; and (v) preregister blinding thresholds and analysis plans. These practices operationalize sham quality as measurable outcomes that can reduce trial variability and strengthen cross-study synthesis in mechanistic and clinical research.

## 1 Introduction

### 1.1 Overview of transcranial direct current stimulation (tDCS)

Transcranial direct current stimulation (tDCS) applies low-amplitude constant current (typically 1–2 mA) through scalp electrodes to modulate cortical excitability by shifting neuronal resting membrane potentials (Nitsche and Paulus, 2000; Stagg and Nitsche, 2011). Despite widespread adoption across cognitive neuroscience (Reinhart et al., 2017), motor rehabilitation (Buch et al., 2017), and psychiatry (Brunoni et al., 2012), outcomes demonstrate considerable variability, with inconsistent replication and limited reliable effects (Horvath et al., 2015; Medina and Cason, 2017). Contributing factors include heterogeneity in stimulation parameters (electrode montage, current intensity, duration) (Chase et al., 2020), individual neuroanatomy and physiology (Li et al., 2015; Kim et al., 2014), task characteristics (Rizvi et al., 2023), and critically, the validity of sham/control conditions (Fonteneau et al., 2019).

### 1.2 Importance of sham controls in tDCS research

Sham stimulation serves as the foundational placebo control in tDCS research, intended to replicate sensory experiences (e.g., tingling and itching at electrode sites) (Ambrus et al., 2012) while minimizing or avoiding neuromodulatory effects (Gandiga et al., 2006). It serves dual functions: masking sensory cues to preserve participant and experimenter blinding and providing a physiologically inert baseline against which active effects can be measured, though this assumption has been questioned (Neri et al., 2020). Conventional sham designs employ brief current ramps (e.g., 30-s fade-in/fade-out) to mimic electrode-associated sensations at the onset of the session, then deliver minimal or zero current for the remainder of the session (Nitsche et al., 2008). This approach, widely adopted since early tDCS trials, rests on two assumptions: that conventional sham protocols achieve effective blinding throughout sessions and that transient peripheral stimulation produces negligible cortical modulation while maintaining sensory plausibility (Turner et al., 2021; Poreisz et al., 2007).

However, accumulating evidence challenges both assumptions. Time-resolved blinding probes indicate that perceptual divergence can emerge during stimulation even when end-of-study guesses approach chance levels (Turner et al., 2021; Greinacher et al., 2019; see Section 10). In addition, electrophysiological and neuroimaging readouts suggest that some sham implementations may not be fully physiologically inert (Nikolin et al., 2018; Wörsching et al., 2017; Park et al., 2025; see Electrophysiological Assessments and Functional MRI/fNIRS sections). These findings motivate closer evaluation of sham protocols with respect to blinding efficacy, test–retest reliability, and physiological inertness.

### 1.3 Purpose and scope of the review

This review synthesizes evidence on test–retest reliability and the blinding efficacy of sham tDCS protocols published between January 1, 2010, and August 31, 2024, with a targeted post-search update (September 2024–September 2025) to capture key methodological advances directly informing sham design, blinding assessment, or physiological inertness. This period spans foundational sham-control practices and their evolution alongside HD montages (Richardson et al., 2014; Kuo et al., 2013), device-locked randomization (Kold and Graven-Nielsen, 2023; Jog et al., 2025), and refined sensory-matching approaches (Neri et al., 2020; Li et al., 2022). We focus on empirical studies that directly assess sham performance through behavioral and neurophysiological endpoints, blinding probes, and physiological markers of inertness. Where directly relevant to sham design considerations, we include selected comparative evidence from transcranial alternating current stimulation (tACS) (Sheffield et al., 2022; Soleimani et al., 2023), particularly regarding shared challenges in sensory masking and blinding control procedures. These inclusions are limited to methodological issues and do not imply physiological equivalence between modalities. Concerns regarding methodological factors, including sham reliability, blinding efficacy, and sample size, directly affect effect size estimation, internal validity, and cross-study synthesis (Fonteneau et al., 2019; Minarik et al., 2016) and are therefore not ancillary to tDCS research but central to interpreting the existing literature and designing robust future trials. The review then outlines specification-grade recommendations and an implementation checklist to operationalize sham quality as a measurable design parameter, rather than treating it as an assumed feature.

## 2 Methods

### 2.1 Review design and reporting framework

**Figure 1:**
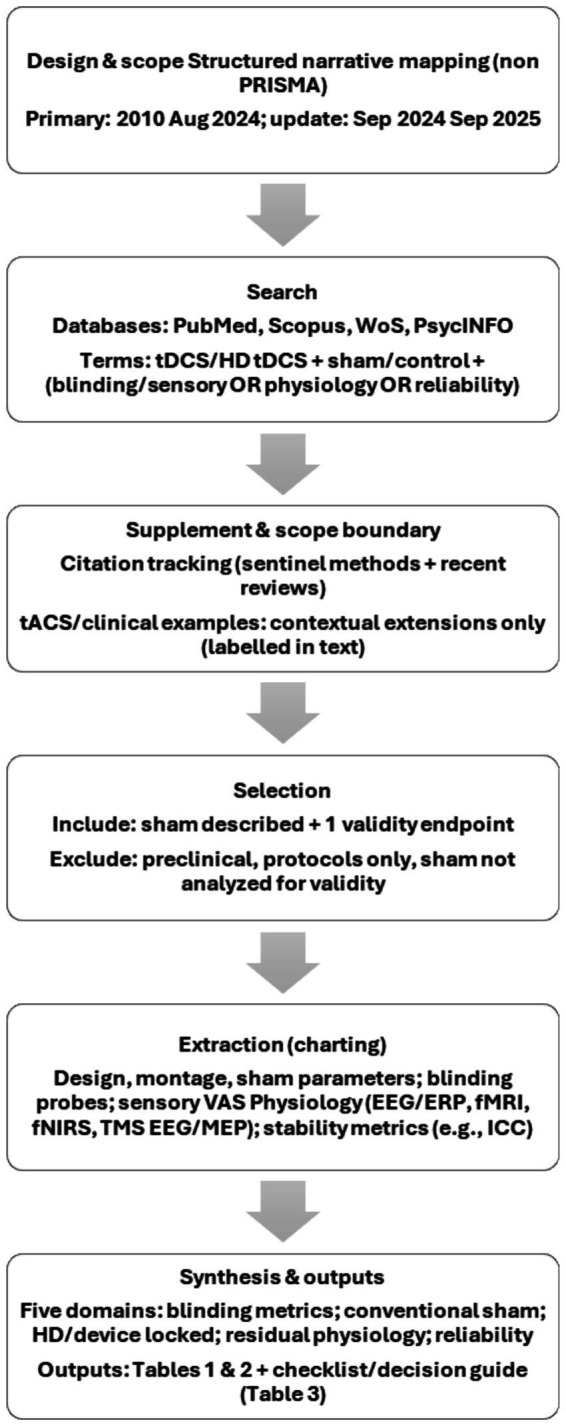
Narrative mapping workflow for sham tDCS methodology (non-PRISMA; workflow summary, not a PRISMA flow diagram).

This study is a structured narrative scoping review designed to map and critically appraise sham control methods and blinding assessment practices in tDCS research, with emphasis on physiological inertness, sensory matching, and endpoints of reproducibility and reliability. Given the substantial heterogeneity in sham implementation, blinding metrics, and outcome domains, precluding meaningful quantitative synthesis, we used a structured narrative mapping approach rather than meta-analysis (Chase et al., 2020; Woods et al., 2016). As many included papers were methodological rather than clinical trials, formal risk-of-bias tools were not applied. This review used a structured, non-exhaustive search strategy to identify illustrative methodological exemplars, rather than to comprehensively catalog all eligible studies. Although we used predefined eligibility criteria and multi-database searches to ensure breadth and conceptual coverage, this process was not designed as a fully systematic review aimed at reproducibly enumerating all eligible records. Accordingly, this manuscript does not include a PRISMA or PRISMA-ScR flow diagram instead, a narrative workflow summary is provided (Figure 1).

### 2.2 Eligibility criteria

We included peer-reviewed studies that met all of the following criteria: (i) human participants; (ii) tDCS, including conventional and high-definition tDCS (HD-tDCS); (iii) a sham or placebo-like control condition with sufficient procedural description (e.g., ramp sham, brief-stimulation variants, low-intensity “active” sham, current-steered/multipolar sham) (Ambrus et al., 2012; Neri et al., 2020; Nitsche et al., 2008; Richardson et al., 2014); and (iv) at least one sham-validity endpoint, including blinding assessment (including time-resolved probes) (Turner et al., 2021; Greinacher et al., 2019; Wallace et al., 2016), physiological evidence bearing on inertness (e.g., EEG/ERP, fMRI, fNIRS, TMS-EEG/MEPs) (Nikolin et al., 2018; Wörsching et al., 2017; Park et al., 2025), and/or reliability/reproducibility outcomes (e.g., intraclass correlation coefficient [ICC], within-subject stability) (Wörsching et al., 2017; Abdelmotaleb et al., 2025). We excluded preclinical (animal/*in vitro*) studies, protocols without results, and reports in which sham was present but not evaluated for blinding, inertness, reliability, or sensory equivalence.

Scope boundary and contextual extensions. We did not conduct dedicated database searches for (i) clinical cohorts (e.g., stroke) or (ii) non-tDCS tES modalities (e.g., tACS). When such studies are discussed, they are used only as contextual extensions to illustrate sham/blinding principles directly relevant to tDCS (e.g., sensory masking strategies, device-locking/auditability, or altered somatosensory thresholds affecting sham credibility). These instances are explicitly labeled in the synthesis as [Contextual extension, outside primary search scope] (Sheffield et al., 2022; Soleimani et al., 2023; Abdelmotaleb et al., 2025) to distinguish them from primary tDCS sham-focused evidence.

### 2.3 Timeframe and scope

The primary coverage window spanned January 1, 2010, to August 31, 2024, chosen to include foundational work shaping modern sham practices and to capture later developments such as HD/multipolar approaches (Richardson et al., 2014; Kuo et al., 2013; Woods et al., 2016), programmable device-locked systems (Greinacher et al., 2019; Wallace et al., 2016; Gbadeyan et al., 2016), and time-resolved blinding assessment protocols (Turner et al., 2021; Greinacher et al., 2019). In addition, we performed a targeted post-search update (September 2024–September 2025) restricted to advances directly informing sham methodology, blinding integrity, or physiological inertness. These additions are explicitly labeled as post-search updates in the text (and tables where applicable).

### 2.4 Information sources and search strategy

We searched PubMed/MEDLINE, Scopus, Web of Science, and PsycINFO for the period January 1, 2010, through August 31, 2024, using Boolean combinations of stimulation terms (tDCS/HD-tDCS), sham/control terminology, and at least one of the following domains: blinding/sensory integrity, reliability/reproducibility, or physiological inertness/objective measures. The post-search update was completed in September 2025. We supplemented database searches with backward/forward citation tracking from sentinel methodological papers (Ambrus et al., 2012; Gandiga et al., 2006; Nitsche et al., 2008) and recent systematic reviews (Bikson et al., 2016; De Smet et al., 2021). Full database-specific search strings are provided in the Supplementary materials. Post-search updates were incorporated transparently and are reported separately from the primary scoping dataset. Study identification and inclusion were guided by conceptual relevance to sham methodology rather than by an exhaustive catalog.

### 2.5 Study selection process

Records retrieved from database searches and citation tracking were reviewed for relevance to the predefined thematic domains. Selection focused on studies providing substantive methodological detail regarding sham implementation, blinding assessment, physiological inertness, or reliability endpoints. Articles that did not directly inform these domains were excluded from the narrative synthesis.

### 2.6 Data charting and extraction

We extracted: study design (parallel/crossover), sample size, montage configuration (bipolar vs. HD/multipolar) (Richardson et al., 2014; Kuo et al., 2013), sham protocol specification (ramp parameters; brief-stimulation variants; device-locked implementation; residual monitoring currents where reported) (Nikolin et al., 2018; Wallace et al., 2016; Gbadeyan et al., 2016; Arroyo-Fernández et al., 2021), stimulation dose, blinding assessment method and timing (time-resolved vs. end-of-study guess) (Turner et al., 2021; Greinacher et al., 2019; Wallace et al., 2016), sensory reporting (VAS; adverse effects), primary outcome modality (behavioral/physiological), and key findings related to blinding efficacy, physiological inertness, and/or test–retest reliability (Nikolin et al., 2018; Wörsching et al., 2017; Park et al., 2025; Abdelmotaleb et al., 2025).

### 2.7 Synthesis approach and quality signals

Evidence was synthesized thematically across five domains: (1) blinding metrics and their validity (Turner et al., 2021; Greinacher et al., 2019); (2) conventional ramp shams and limitations (Ambrus et al., 2012; Nitsche et al., 2008); (3) HD/multipolar and device-locked innovations (Neri et al., 2020; Richardson et al., 2014); (4) evidence of residual physiological effects under sham (Nikolin et al., 2018; Wörsching et al., 2017; Park et al., 2025); and (5) test–retest reliability across behavioral and neurophysiological measures (Wörsching et al., 2017; Abdelmotaleb et al., 2025). Where patterns converged across high-quality studies, we highlight consensus findings (Chase et al., 2020; Fonteneau et al., 2019; De Smet et al., 2021). Where methods diverged, we noted sources of heterogeneity (e.g., ramp duration, current intensity, assessment timing) that limit generalization (Chase et al., 2020; Wallace et al., 2016). We did not apply formal risk-of-bias tools (e.g., RoB 2; Sterne et al., 2019) because many included papers are methodological rather than clinical trials. Instead, we evaluated fit-for-purpose quality signals relevant to sham validity, including clarity of sham implementation and device-lock/randomization procedures, adequacy and timing of blinding assessment, control for sensory confounds, and mitigation of practice effects in multi-session designs. Therefore, these quality signals were used to guide interpretation and do not constitute a formal risk-of-bias rating. Studies with pre-registration (Nosek et al., 2018), CONSORT-aligned reporting (Schulz et al., 2010; Cuschieri, 2019), or triple-blind procedures (Loreti et al., 2023) were weighed more heavily in interpretation. Because these signals are not commensurate (e.g., time-resolved probing vs. device-locking vs. sensory reporting) and are inconsistently available across studies, we did not compute an additive score. Instead, we provide a pragmatic three-level summary (●/●●/●●●) reflecting diagnostic/audit rigor and reporting transparency specific to sham validity (Table 1).

**Table 1:** Blinding efficacy in sham tDCS (2012–2025).

Study (ref)	Design (sessions)	*N*	Montage	Active dose	Sham specification	Blinding assessment	Blinding outcome	Quality signals	Quality	Key finding (brief)
Ambrus et al. (2012)	Randomized, double-blind, within-subject (crossover); 3 sessions (anodal, cathodal, sham); ≥4-day interval.	36	Anodal: anode C3/cathode left supraorbital; cathodal: reversed polarity; sham: polarity randomized; carbon rubber electrodes in saline-soaked sponges (size NR).	1 mA; 10 min total (including 20 s ramp-up and 10 s ramp-down).	1 mA; 60 s total (including 20 s ramp-up, 30 s stimulation, 10 s ramp-down).	Time-resolved perception ratings (every 1.75 min) + post-session questionnaire; real/placebo guess + confidence rating.	Mixed	TR, Rpt, BlindLvl	●●	Blinding successful in participants; failed in investigators; cutaneous sensations persisted in both sham and active conditions.
O’connell et al. (2012)	Andomized, double-blind, within-subject (crossover), sham-controlled; 2 sessions (active, sham); ≥2-week interval.	100	Anode left motor cortex/cathode right supraorbital; rubber electrodes in saline-soaked sponges (35 cm^2^).	2 mA; 20 min total (including 5 s ramp-up and ramp-down).	2 mA; ~30 s total (including 5 s ramp-up; device switched off after 30 s).	Post-session real/placebo guess + confidence rating; skin redness assessed by researcher.	NE	Sensory/AE reporting, Rpt, BlindLv	●●	Blinding inadequate at 2 mA; participants identified condition above chance; skin redness unmasked assessors.
Gbadeyan et al. (2016)	Double-blind, sham-controlled, within-subject (crossover); 2 visits (active, sham); ≥3-day interval; between-subject montage groups (conventional vs. HD).	60	Conventional: anode F4/cathode supraorbital; rubber electrodes in saline-soaked sponges (anode 5 × 7 cm^2^; cathode 10 × 10 cm^2^); HD: centre anode (Ø2.5 cm) + ring cathode (Ø9.2/11.5 cm); conductive rubber electrodes with gel.	1 mA; 20 min total (including 10 s ramp-up; ramp-down NR).	1 mA; ~20 s total (including 10 s ramp-up and 10 s stimulation; ramp-down NR).	End-of-session guess (participant + researcher); skin redness assessed by researcher; adverse effects rating; unpleasantness/pain VAS (repeated measures).	S	Sensory/AE reporting, Roles, DL, Rpt, BlindLvl	●●●	Participant and investigator guesses ≈ chance; HD-tDCS better tolerated with comparable unpleasantness for active vs. sham; conventional tDCS showed greater unpleasantness in active vs. sham; sensations tracked over time.
Wallace et al. (2016)	Randomized, double-blind, sham-controlled, within-subject; 2 sessions (active, sham); ≥7-day interval; between-subject age groups (young *n* = 34, older *n* = 30).	64	Anode F8/cathode Fp1; rubber electrodes in saline-soaked sponges (35 cm^2^).	2 mA; 30 min total (including 30 s ramp-up and ramp-down).	2 mA; ~2 min total (including 30 s ramp-up, 1 min stimulation, 30 s ramp-down) + low-intensity pulses (~110 μA every 550 ms).	End-of-study guess (participant + researcher); comfort VAS.	Mixed	Sensory/AE reporting, Roles, DL, Rpt, BlindLv	●●●	Participants showed a trend toward above-chance identification in session 2; researcher identified condition above chance overall; sham included low-intensity impulse current; comfort lower at stimulation onset and differed by age
Loo et al. (2018)	Randomized, double-blind, sham-controlled, parallel-group; 20 sessions over 4 weeks; optional open-label extension + taper phase.	130	Anode F3/cathode F8; rubber electrodes in saline-soaked sponges (7 × 5 cm^2^).	2.5 mA; 30 min total (ramp-up/down NR; weekday sessions).	0.034 mA (continuous); ~30 min (10 s ramp-up to 1 mA + 60 s ramp-down at start; 60 s ramp-up to 0.5 mA + 60 s ramp-down at 10 or 20 min).	End-of-trial guess (participant + researcher); treatment expectancy questionnaire (pre-treatment); adverse effects rating.	S	Sensory/AE reporting, Roles, Rpt, BlindLvl	●●●	End-of-trial guesses not associated with actual assignment; sham delivered continuous low-intensity current (0.034 mA), suggesting potential biological activity.
Nikolin et al. (2018)	Randomized, single-blind, parallel-group; single-session; two experiments; five conditions across experiments (2 mA, 1 mA, sham 0.034 mA, sham 0.016 mA, Off).	100	Anode F3 (left DLPFC)/cathode F4 (right DLPFC); rubber electrodes in saline-soaked sponges (16 cm^2^).	1 mA or 2 mA; 15 min total (including 30 s ramp-up and ramp-down).	Sham1 (Eldith): 2 mA; ~90 s total (including 30 s ramp-up, 30 s stimulation, 30 s ramp-down) + 0.016 mA background; Sham2 (Soterix): 1 mA; ~70 s total (including 10 s ramp-up and 60 s ramp-down) + 0.034 mA background; Off: 0 mA.	End-of-session guess (active vs. sham).	Mixed (blinding compromised; Off condition more easily identified)	BlindLvl	●●	Off condition identified above chance; residual-current shams more similar to active sensations than Off and produced measurable EEG effects; 0.034 mA sham likely not biologically inert.
Greinacher et al. (2019)	Double-blind; counterbalanced, within-subject crossover; 2 visits (active, sham); ≥24 h washout; NeuroConn “study mode” (coded protocols for double-blinding).	32	Anode C3 (left M1); cathode right forehead; 5 × 7 cm rubber electrodes in saline-soaked sponges.	1 mA; 10 min total (including 30 s ramp-up and ramp-down).	1 mA; 80 s total (including 30 s ramp-up + 20 s stimulation + 30 s ramp-down); then 110 μA impedance-check pulses every 550 ms.	Online time-resolved guess (“stimulation on?”) every 30 s + confidence rating; End-of-study sham guess (session identification + confidence).	NE	TR, Sensory/AE reporting, DL, Rpt, BlindLvl	●●●	End-of-study session identification (sham vs. active) above chance; Time-resolved probes show divergence emerging immediately after sham ramp-down and persisting throughout stimulation period.
Neri et al. (2020)	Within-subject, randomized, counterbalanced crossover; 4 sessions (real Bifocal, Bifocal-Sham, real Multifocal, ActiSham); ≥7 ± 3 days washout.	14	Bifocal: anode C3 (left M1); cathode Fp2; 35 cm^2^ rubber electrodes in saline-soaked sponges; Multifocal (real): 4-channel montage targeting left M1; Ø20 mm Ag/AgCl gel electrodes; ActiSham: 6-channel montage (model-driven shunting) targeting left M1; Ø20 mm Ag/AgCl gel electrodes	Bifocal: 2 mA; 15 min total (including 30 s ramp-up and ramp-down); Multifocal: multichannel stimulation (max ~2 mA per electrode); 15 min total (including 30 s ramp-up and ramp-down).	ActiSham: multichannel stimulation; 15 min total (including 30 s ramp-up and ramp-down; model-driven shunting to minimize cortical E-field); Bifocal-Sham: 2 mA; FISSFO protocol (brief stimulation after ramp-up; duration NR).	End-of-stimulation real/sham guess; Pain/annoyance/intensity VAS; Adverse-effects questionnaire; scalp localization (site and focal vs. distributed perception); Operator blinding not possible (different electrode types).	Mixed	Sensory/AE reporting, Roles, Rpt	●●	ActiSham improved sensory plausibility (matched Real multifocal sensations) while minimizing corticospinal effects; Bifocal stimulation more easily discriminated from sham.
Arroyo-Fernández et al. (2021)	Randomized, triple-blind, sham-controlled, parallel-group trial; 3 arms (active tDCS, sham, control); 5 sessions over 2 weeks.	80	Anode C3 (left M1); cathode Fp2 (right supraorbital); 25 cm^2^ circular sponge electrodes in saline solution.	2 mA; 20 min total (including 30 s ramp-up and ramp-down).	2 mA; FISSFO protocol (30 s ramp-up + immediate ramp-down at beginning and end; no stimulation between).	End-of-trial questionnaire (participants and therapist; assessed for active vs. sham only).	S	BI, Roles, Rpt, BlindLvl	●●●	Blinding successful (James’/Bang’s indices); Therapist blinding less robust than participants; High proportion of “do not know” responses.
Turner et al. (2021)	Double-blind, counterbalanced, within-subject crossover; 2 sessions (active, sham); ≥24 h washout.	32	Anode C3 (left M1); cathode right forehead; 5 × 7 cm carbon rubber electrodes in saline-soaked sponges.	2 mA; 10 min total (including 30 s ramp-up and ramp-down).	2 mA; 80 s total (including 30 s ramp-up + 20 s stimulation + 30 s ramp-down).	Time-resolved probes every 30 s + confidence rating; End-of-session adverse-effect ratings; End-of-study sham guess.	NE	TR, DL, Rpt, BlindLvl	●●●	Greater active–sham divergence at 2 mA; End-of-study guess poorly reflects blinding; Time-resolved probes detect unblinding.
Li et al. (2022)	Randomized, double-blind, counterbalanced crossover; 3 sessions (anodal, cathodal, sham); ≥1 week washout.	28	HD 4 × 1; center C3 or C4 (M1); returns at FC1/FC5/CP5/CP1 or FC2/FC6/CP6/CP2; Ag–AgCl ring electrodes.	1 mA; 20 min total (including 30 s ramp-up and ramp-down).	1 mA; ~60 s total (30 s ramp-up + 30 s ramp-down at beginning; repeat ramp-up at end; ramp-down NR).	Post-session binary guess (active vs. sham) + adverse-effect ratings.	S	Sensory/AE reporting, Rpt, BlindLvl	●●	AE ratings largely comparable across conditions; No difference in blinding guesses (successful blinding); No systematic unblinding across sessions.
Stanković et al. (2022)	Secondary analysis of 4 within-subject crossover sham-controlled memory studies; counterbalanced; 2–3 visits; 1–2 week intervals.	83	Anode P3/P4/F3/F4 (PPC/DLPFC); cathode contralateral cheek; 5 × 5 cm rubber electrodes in saline-soaked sponges.	1.5–1.8 mA; 20 min total (including 30 s ramp-up and ramp-down).	20 min (ramp-up immediately followed by ramp-down at beginning and end; no stimulation between; ramp intensity not reported).	End-of-study sham guess.	S	Rpt	●	Correct sham guess did not moderate memory outcomes; Guessing at/near chance; End-of-study guess may not capture unblinding; no impact on objective (computerized) measures.
Sheffield et al. (2022)	Parallel, multisite; repeated stimulation (up to 11 sessions over ~2 weeks); 2 experiments.	1,019; sham *n* = 503; active: tDCS *n* = 160, tACS *n* = 190, tRNS *n* = 166	tDCS: anode F3; cathode AF8; 3.14 cm^2^ circular Ag/AgCl electrodes.	1.25 mA; 30 min total.	1.25 mA; FSF protocol (Exp1: 30 s at start + end; Exp2: 20 s at start only; no stimulation between).	End-of-study active vs. sham guess + confidence rating; AE reports at start and end of each session (severity + attribution to stimulation).	Mixed (varies by modality)	Rpt	●●●	Greater cutaneous sensation associated with poorer blinding; Multisite variability in blinding and AE reports; Blinding reduced for tDCS/tACS vs. tRNS; Repeated sessions decreased AE reporting over time.
El Jamal et al. (2023)	Pooled dataset; multisession HD-tDCS; 3,046 sessions (2,329 active, 717 sham); Mostly double-blind.	292; Mixed clinical diagnoses.	HD-tDCS (4 × 1 configuration); Ag/AgCl electrodes (4 mm radius) in electroconductive gel.	1–4 mA (center electrode; total current up to 10 mA); 20–30 min total (including 30 s ramp-up and ramp-down); Varied across parent studies.	Active-intensity; bimodal ramp (30 s ramp-up/down at start and end; no stimulation between); 20–30 min total.	End-of-session active vs. sham guess (participants); study team guess (session 5 & final); AE reports (participants) + skin redness assessed by study team.	S	DL, BlindLvl	●●●	Comparable tolerability and effective blinding (participants and study team); Some sensations more frequent after sham (recency effect) and at lower active intensities
Turi et al. (2019)	Pre-registered, double-blind, Parallel-group; Single session; Multisite (3 labs).	192 (real = 96; FSF sham = 96); (*n* = 64/site).	Anode F3 (4 × 4 cm); cathode right supraorbital (7 × 5 cm); Rubber electrodes with conductive paste.	1 mA; ~21 min total (20 min stimulation + 30 s ramp-up and ramp-down).	1 mA; 20 min (30 s ramp-up + 15 s stimulation + 30 s ramp-down at session start only).	Post-session discomfort rating; Post-session real vs. sham guess.	NE	Sensory/AE reporting, Rpt, BlindLvl	●●●	Condition guessed above chance; Greater discomfort with real tDCS; FSF does not ensure complete blinding
Kold and Graven-Nielsen (2023)	Randomized, double-blind, sham-controlled, parallel-group; 4 arms (Active-tDCS, Sham-tDCS, Pain-Active-tDCS, Pain-Sham-tDCS); 3 sessions (24 h apart).	80	HD multichannel: anodes C3 and F3; cathodes AF3, CP1, FC1, FC5, CP5; 3.14 cm^2^ Ag/AgCl gelled electrodes.	2 mA per anode (HD multichannel); 20 min total (including 30 s ramp-up and ramp-down).	2 mA; 20 min (30 s ramp-up + 19 min no stimulation + 30 s ramp-down).	Post-session active vs. sham guess; Sham-trust index.	Mixed	DL, Rpt, BlindLvl	●●	Mixed blinding (device-based sham); Sham-trust index comparable across groups; Accuracy higher in active-tDCS group and on first session.
Loreti et al. (2023)	Randomized, triple-blind, sham-controlled, parallel-group trial; 10 sessions (~2 weeks; 5 consecutive + 2-day break + 5 sessions).	35 (active 17; sham 18)	Anode C3 (M1); cathode right supraorbital; 35 cm^2^ electrodes in saline-soaked sponges	2 mA; 26 min total stimulation (13 min + 13 min; 20 min break between blocks); 10 sessions; ramp parameters NR.	2 mA; ~1 min total stimulation (≈30 s at start of each 13 min block; no stimulation between); 13:20:13 protocol; ramp parameters NR.	Participant belief (active vs. sham; timing NR); Post-session sensory/adverse-effect reporting.	S (authors report blinding maintained; Both groups predominantly believed ‘device on’).	Sensory/AE reporting, Roles, DL, Rpt, BlindLvl	●●●	Triple-blind, multi-session design with device-programmed sham; AE monitoring (including skin irritation) highlights importance of tolerability assessment.
Jog et al. (2025)	Parallel; Double-blind; 12 sessions	71 (active = 40; sham = 31).	MRI-guided (structural) frameless stereotaxic neuronavigation; HD-tDCS (4 × 1 configuration) targeting left DLPFC; 5 electrodes (2 × 2 cm).	2 mA; 20 min (including ~30 s ramp-up/down).	20 min (ramp-up/down + 0.065 mA remainder).	end-of-study treatment allocation guess (participants and assessors); post-session discomfort and AE reporting.	S	DL, Roles, Rpt, BlindLvl	●●●	Allocation guess collected from participants and assessors; no difference in guesses between groups (successful blinding); low-intensity sham (0.065 mA).

Blinding outcome: S, successful; NE, not effective; Mixed, depends on role/timepoint/measure; NR, not reported. Quality signals: TR, time-resolved blinding probes; BI, Bang/James (or equivalent) blinding index; Sensory/AE reporting, sensory ratings and/or adverse-effect/tolerability measures (any scale type); Roles, blinding assessed across roles (participant/therapist/assessor/operator) or triple-blind; DL, device-locked/programmed allocation and/or audit trail; Rpt, key sham parameters reported (otherwise NR); BlindLvl, blinding level explicitly stated (single/double/triple). BlindLvl is reported for transparency and is not treated as an additive score. AE, adverse events; EEG, electroencephalography; RCT, Randomized Clinical Trial; FSF, Fade-in/short stimulation/fade-out; MRI, Magnetic Resonance Imaging; tDCS, transcranial Direct Current Stimulation; tACS, transcranial Alternating Current Stimulation; tRNS, transcranial Random Noise Stimulation; HD-tDCS, High-Definition transcranial Direct Current Stimulation; FISSFO, Fade-In Short Stimulation Fade-Out; DLPFC, Dorsolateral Prefrontal Cortex; PPC, Posterior Parietal Cortex. Quality (●/●●/●●●): pragmatic summary of diagnostic/audit rigor and reporting transparency (not a clinical trial risk-of-bias score). Sessions terminology: “visits” = sessions/participant (e.g., crossover); “sessions” = treatment schedule (e.g., 10–20 interventions); “total sessions” = dataset-level total when reported. Quality signals are reported as a comma-separated set: each code indicates that the feature was assessed and/or transparently reported; absence indicates not assessed or not reported. Active dose refers to the intended plateau intensity/duration of the active condition(s) used in that study; where not reported, it is coded NR. In FSF shams, the ramp peak often matches the nominal active intensity but only for brief segments and should not be interpreted as an equivalent time-integrated dose.

## 3 Results overview

The included studies mapped in this review span two complementary domains: (i) blinding efficacy and sensory integrity of sham tDCS protocols across single- and multi-session designs (summarized in Table 1), and (ii) test–retest reliability and stability of behavioral and neurophysiological outcomes under sham conditions (summarized in Table 2). The tables provide a structured overview of study design, sham specification, assessment methods, and key findings, which are synthesized narratively below to highlight recurring methodological patterns, limitations, and advances.

**Table 2:** Stability across sessions under sham tDCS (2012–2025).

Study (ref)	Design (sessions)	*N*	Outcome modality	Active dose	Sham specification	Reliability metric	Quality signals	Quality	Key finding (brief)
Wörsching et al. (2017)	Double-blind; sham-controlled; parallel-group (active vs. sham); 3 sessions (≥7-day intervals).	20 (all males)	Resting-state fMRI connectivity (ROI-based, ICA).	2 mA; 20 min (including 15 s ramp-up/down).	20 min (ramp-up/down); no sustained stimulation between, with low-threshold pulses; intensity NR; protocol per (Palm et al., 2013).	Test–retest reliability: ICC (ROI-based and ICA-based analyses; active and sham).	RS, ICC, Rpt, BlindLvl	●●●	Sham-related connectivity changes observed at group level; low test–retest reliability for active tDCS; sham reliability comparable to baseline.
Nikolin et al. (2018)	Single-blind, parallel-group (two-stage allocation); single session; 5 arms (2 mA, 1 mA, sham 0.034 mA, sham 0.016 mA, Off).	100 (20/arm)	Behavioral (working memory performance) + EEG/ERP (P3 amplitude).	1 mA; 15 min (bifrontal); 2 mA; 15 min (bifrontal).	0.034 mA “sham”; 0.016 mA “sham”; Off; 0 mA.	NR; EEG differences vs. Off indicate residual-current sham not inert.	Neutral	●●	Residual-current sham altered ERP vs. Off (unplugged); sham may not be biologically inert and can affect neural activity
Stanković et al. (2022)	Secondary analysis of 4 within-subject crossover sham-controlled memory studies; counterbalanced; 2–3 visits; 1–2 week intervals.	83	Behavioral (associative and working memory tasks); outcomes: correctly recalled pairs, accuracy, reaction time.	1.5–1.8 mA; 20 min total (including 30 s ramp-up and ramp-down).	20 min (ramp-up immediately followed by ramp-down at beginning and end; no stimulation between; ramp intensity not reported).	NR	RS, Rpt	●	Correct sham guess did not moderate objective memory outcomes (AM/WM); no evidence that sham-related beliefs influenced outcomes across sessions
Hsu et al. (2023)	Randomized, double-blind, sham-controlled, parallel-group design; 20 sessions (2/day × 10 days over 2 weeks).	27 (active = 13; sham = 14)	Clinical (motor recovery scales: FMA-UE, ARAT, FMA-LE); neuroimaging (rs-fMRI connectivity, ROI-based); neurophysiological (motor-evoked potentials, TMS-derived).	2 mA; 20 min total (including 30 s ramp-up and ramp-down).	Identical setup; 20 min total (including 30 s ramp-up; current ceased after 2 min; remainder off).	NR (no formal reliability/test–retest metric); longitudinal sham arm included but not analyzed for stability.	Rpt, BlindLvl	●●	Extended multi-session schedule; no sham-specific stability or reliability analysis reported; both active and sham groups showed longitudinal motor improvements, with significantly greater gains in the active tDCS group
Abdelmotaleb et al. (2025)	Within-subject; sham-only; 2 visits; ≥1 week interval.	20	Behavioral (object–location memory task performance); neuroimaging (task-based fMRI activation, ROI-based/TRR analysis).	No active stimulation (sham-only)	2 mA; 40 s total (including 10 s ramp-up, 20 s stimulation, 10 s ramp-down).	ICC (behavioral performance and task-based fMRI activation).	ICC, RS, Rpt	●●●	Behavioral and task-based fMRI measures showed moderate-to-good test–retest reliability (ICC), supporting reproducibility across sessions under sham conditions

Signals: RS = repeated-session design enabling across-session stability inference; ICC = intraclass correlation reported; Neutral = explicit no-current comparator (e.g., unplugged/Off); DL/Rpt as in Table 1. Signals are reported as a comma-separated set; RS refers specifically to ≥2 sham sessions per participant (enabling across-session stability inference). fMRI, functional Magnetic Resonance Imaging; ICA, Independent Component Analysis; ROI, Region of Interest; tDCS, transcranial Direct Current Stimulation; AM, Associative Memory; WM, Working Memory; OLM, Object-location memory; FMA-UE, Fugl-Meyer Assessment – Upper Extremity; ARAT, Action Research Arm Test, FMA-LE, Fugl-Meyer Assessment – Lower Extremity; rs-fMRI, resting-state functional Magnetic Resonance Imaging.

## 4 Technical foundations of sham tDCS

### 4.1 Biophysical basis of tDCS and sham stimulation

Sham tDCS aims to replicate cutaneous perceptions (tingling, itching, warmth) generated by peripheral activation during current onset while minimizing intracranial electric fields that could influence cortical activity (Poreisz et al., 2007; Fertonani and Miniussi, 2016). The design challenge is biophysical: scalp sensations arise from activation of mechanoreceptors and nociceptors in the dermis during current ramps and are typically strongest near electrode edges where current density is highest (Boekholdt et al., 2021). These perceptions often diminish within the first ~20–120 s as peripheral adaptation occurs (Turner et al., 2021; Greinacher et al., 2019; O’connell et al., 2012), creating a temporal window in which sham and active stimulation can diverge if the sham current ceases entirely.

Conventional FSF shams exploit this adaptation window by ramping the current to the target intensity (typically 1–2 mA over ~10–30 s) and then ramping it down, on the premise that participants may not reliably detect the absence of sustained stimulation (Gandiga et al., 2006). However, this premise can be challenged by several factors: (i) inter-individual variation in skin properties and sensory responses (Ezquerro et al., 2017); (ii) task demands or contextual cues that can refresh attention to scalp sensations during the session (Greinacher et al., 2019); and (iii) modeling studies indicating that even brief ramps can generate measurable intracranial electric fields (on the order of sub-volt-per-meter) under some montages (Opitz et al., 2015; Huang et al., 2017), particularly for bipolar configurations that direct current more perpendicularly relative to the scalp.

### 4.2 Common sham protocol designs and variations

Sham protocols can be grouped into three broad generations, each addressing limitations of earlier designs while introducing new trade-offs. First-generation approaches employed simple ramp-up/short stimulation/ramp-down (FSF) at session onset (Gandiga et al., 2006; Nitsche et al., 2008). This provides brief sensory masking but does not reproduce the sustained sensory profile associated with prolonged active stimulation (Neri et al., 2020). Second-generation protocols introduced additional ramps (e.g., early and late session) or periodic brief pulses [e.g., every 550 ms (Wallace et al., 2016) or additional ramp-up/down events at 10 or 20 min (Loo et al., 2018)] intended to approximate more sustained sensations. While these variants may improve late-session masking, perceptual divergence can still emerge (Turner et al., 2021; Greinacher et al., 2019; see Section 10 for time-resolved assessment).

Third-generation approaches employ spatial or device-based solutions. HD-tDCS montages (particularly 4 × 1 ring configurations) (Kuo et al., 2013; Woods et al., 2016) constrain the spatial spread of intracranial electric fields, producing more focal cortical stimulation compared to conventional pad-based approaches (Dmochowski et al., 2011). Additionally, compared to conventional tDCS, HD-tDCS has been associated with modulation of sensory thresholds and modest effects on thermal and cold pain perception (Borckardt et al., 2012). ActiSham exemplifies this approach, delivering controlled, multifocal currents that mimic sensations of transcranial stimulation without significant modulation of corticospinal excitability (Neri et al., 2020). Validation studies indicate that ActiSham can approximate the sensory profile of active 4 × 1 HD-tDCS without measurable changes in TMS-MEP, although some configurations (e.g., bifocal variants) may be more detectable due to higher local current density (Neri et al., 2020).

Parallel advances in device-programmed stimulation systems (e.g., Soterix Mini-CT, NeuroConn DC-Stimulator Plus) embed sham protocols within device-controlled algorithms, reducing manual switching that can compromise experimenter blinding (Wallace et al., 2016; Gbadeyan et al., 2016). These systems often include features such as impedance monitoring and automated delivery, enabling verification of protocol fidelity in complex multi-region trials (Kold and Graven-Nielsen, 2023). Some devices implement hybrid sham approaches that combine ramp-based protocols with low-level background currents while remaining well below typical stimulation doses (Nikolin et al., 2018).

Alternative control strategies, such as stimulating an unrelated region or applying opposite polarity, are sometimes used as “active controls.” However, these should not be considered equivalent to true sham conditions because they involve neuromodulatory current and do not isolate placebo contributions (Woods et al., 2016). Other studies have proposed active control conditions using low-intensity stimulation (e.g., 0.5 mA) to better match sensory experience, which did not influence inhibitory control task performance (Brunyé et al., 2014). This methodological approach may be particularly valuable in repeated-measures designs, where differences in sensory experience can be more readily detected (Brunyé et al., 2014). Current intensity remains an important practical parameter, as blinding degrades with higher intensities (e.g., 2 mA vs. 1 mA) (Sterne et al., 2019; Loo et al., 2018). In addition, some reports indicate non-linear intensity–perception relationships in HD-tDCS, with sensory experiences not increasing monotonically at higher intensities, potentially reflecting peripheral processes such as nociceptor adaptation or skin responses (El Jamal et al., 2023).

### 4.3 Neural and sensory implications of sham parameters

Three parameter dimensions interact to determine sham quality: electrode configuration, ramp kinetics, and current amplitude. Larger electrodes (25–35 cm^2^) distribute current over a greater surface area, typically reducing peak current density at any single site and thereby attenuating focal cutaneous intensity. However, from a physiological perspective, stimulating a larger skin region may recruit more sensory afferents, potentially enhancing perception through spatial summation. Conversely, smaller high-definition electrodes (<5 cm^2^) concentrate current more intensely near electrode edges, which may increase localized cutaneous sensation while enabling more precise intracranial field shaping (Kuo et al., 2013; Woods et al., 2016). This trade-off is central since conventional bipolar montages distribute current and sensation more broadly but yield less constrained intracranial electric fields, whereas HD configurations concentrate current locally, increasing the likelihood of focal cutaneous intensity while enabling improved cortical focality.

Ramp duration can influence the temporal dynamics of perceived sensation, and some protocols employ extended ramp periods to modulate onset sensations (Loo et al., 2018). Faster ramps (<15 s) may produce more abrupt sensory transients that approximate active stimulation onset but may increase discomfort, particularly at higher intensities such as 2 mA (Ambrus et al., 2012; Poreisz et al., 2007). Published protocols vary widely [e.g., from ~5 s (Loo et al., 2018) to ~60 s (Loo et al., 2018)], which complicates cross-study synthesis.

Finally, the assumption that peripheral nerve stimulation (PNS) contributions are negligible in tDCS warrants empirical evaluation, as sham and low-intensity stimulation may still engage peripheral mechanisms (Neri et al., 2020). Proposed controls, such as topical anesthesia, may attenuate cutaneous afferent activity but can introduce confounding factors (e.g., altered impedance and participant awareness). Evidence increasingly suggests that peripheral contributions play a role in tDCS effects, and separating transcranial from transcutaneous mechanisms remains a key methodological challenge (Huang et al., 2017).

## 5 Reliability and stability of sham responses: designs and metrics

This section addresses the stability of sham-related outcomes across time and sessions, which is conceptually distinct from single-session sham–active contrasts. The ideal design to quantify within-person stability of sham responses is repeated sham sessions under matched conditions (i.e., ≥2 sham sessions per participant), enabling estimation of reliability/consistency (e.g., ICC) and separation of measurement noise from true within-person variability. However, most tDCS sham studies use single sham sessions, which can inform group-level comparability but provide limited evidence about within-person stability across days. Accordingly, we distinguish between (i) metrics for quantifying reliability/stability and (ii) the study designs reported in the current literature that permit such estimation.

In our included corpus, only four studies (Table 2) implemented two or more sham sessions per participant in a way that permitted direct estimation of sham stability/reliability, whereas the remaining studies relied on single-session sham comparisons or other designs that did not support direct estimation of across-session stability.

### 5.1 Quantitative metrics for reliability and stability (what can be estimated, and how)

Reliability and stability are not a single property. At minimum, sham outcome assessment should distinguish (a) rank-order stability (whether individuals preserve their relative performance across sessions) from (b) mean change (whether there is systematic learning, fatigue, or other drift under sham). These can coexist, for example, a task may show strong rank-order stability while also showing systematic practice-related improvement.

Intraclass correlation coefficients (ICC). Behavioral stability under sham is often quantified using ICC, which partitions variance into stable between-subject differences and within-subject variability (Shrout and Fleiss, 1979). Because absolute-agreement and consistency ICCs answer different questions about stability, the ICC model should be specified. In the included literature, ICC estimates under sham vary by task, design, and population, with studies reporting moderate-to-good stability across repeated sessions for behavioral and neurophysiological measures (Wörsching et al., 2017; Abdelmotaleb et al., 2025). Several studies report that sham responses can be comparable to baseline or no-stimulation conditions when procedures are standardized and practice effects are accounted for, although variability depends on task and design (Nikolin et al., 2018; Wörsching et al., 2017).

Agreement-based and within-person indices. When the aim is to understand session-to-session variability rather than rank-order stability, complementary indices are useful, including within-person SD, standard error of measurement, coefficient of variation, and (where appropriate) Bland–Altman limits of agreement. These are especially informative when sham is used to detect subtle active–sham differences and when session timing/state variability is expected.

Internal consistency (within-session reliability). Some outcome measures permit estimation of within-session reliability using split-half approaches or internal consistency (e.g., when aggregating across comparable trials). While not a substitute for across-day stability, such indices help identify “noisy” tasks in which low reliability will limit the detectability of any active effect (Hedge et al., 2018; Duff, 2012).

Equivalence framing. In some contexts, sham evaluation is framed as “no meaningful difference” between sham conditions or between sham and baseline/no-electrode controls (rather than “no statistical difference”). In such cases, equivalence testing (e.g., TOST with predefined margins) can be used to support practical equivalence, provided that margins are justified and pre-specified (see Section 10 for details).

A key interpretive point is that differences in stability between sham and active conditions do not automatically indicate sham failure. For example, higher stability under sham than active stimulation within the same design may reflect true inter-individual variability in tDCS responsiveness rather than instability of the sham condition (López-Alonso et al., 2015). Conversely, substantially lower stability under sham than under baseline/no-stimulation conditions may indicate that sham procedures introduce additional sources of variance (e.g., sensory distraction, expectancy effects, or residual currents).

### 5.2 Study designs that permit (or limit) sham stability across sessions (test–retest reproducibility)

Different designs support different inferences about sham outcomes, especially within-person stability across sessions. The following mapping clarifies what can be inferred about sham stability in the existing literature.

- Repeated sham sessions (ideal for stability). Designs with ≥2 sham sessions per participant under matched conditions are the most direct way to estimate sham stability across sessions (e.g., ICC across sham sessions). These designs also allow explicit modeling of practice effects and state variability.
- Single sham session with pre/post outcomes. Pre/post sham designs can test mean change across time (learning/fatigue, time-on-task, expectancy drift) but do not estimate across-day stability unless sham sessions are repeated.
- Crossover active–sham designs with only one sham session. These designs are powerful for active–sham contrasts but provide limited information about sham stability itself (i.e., within-condition reliability). They are also vulnerable to learning and carryover effects depending on task characteristics and washout interval (Bates et al., 2015; Haikalis et al., 2023).
- Parallel-group sham controls. Parallel designs support group-level comparability but do not permit estimation of within-person stability.
- No-electrode/unplugged baselines versus sham. Where available, no-electrode or unplugged controls help isolate whether the sham procedure itself adds variance (e.g., through cutaneous sensation or device currents) beyond ordinary task repeatability (Nikolin et al., 2018). However, such designs remain uncommon.

This design distinction is central to interpretation: a literature dominated by single-session sham comparisons cannot legitimately be interpreted as providing strong evidence about within-person stability across sessions, even if it supports group-level active–sham contrasts.

### 5.3 Behavioral paradigms commonly used and what they imply for sham stability

Behavioral task performance under sham conditions provides a benchmark for evaluating whether repeated sham exposure introduces learning, fatigue, or other confounds that could mask or mimic active tDCS effects. For sham stability assessment, paradigms are most informative when they show: (i) adequate intrinsic repeatability (in the relevant population), (ii) limited ceiling/floor effects across repeated sessions, and (iii) practice-related changes that can be modeled and separated from stimulation effects (Hedge et al., 2018).

Memory tasks, particularly object–location memory (OLM), have been used in multi-session designs (Abdelmotaleb et al., 2025). Across repeated sessions, these paradigms demonstrate moderate-to-good stability, with early practice-related improvements concentrated in initial learning stages (Abdelmotaleb et al., 2025). Similar stability has been observed for visual perceptual tasks, with consistent performance across sessions, supporting their use in crossover designs when practice effects are accounted for (Learmonth et al., 2017).

Executive function paradigms, including the Eriksen Flanker task, Go/No-Go and stop-signal tasks, and n-back working memory, provide complementary assessment of prefrontal cognitive control (Gbadeyan et al., 2016; Jacobson et al., 2011). These tasks often show moderate stability under sham, but performance improvements across sessions are common, necessitating explicit modeling of learning curves (Abdelmotaleb et al., 2025; Hedge et al., 2018). Standardized computerized task delivery is commonly used in tDCS studies to ensure consistency of administration (Stanković et al., 2022).

Simple reaction time tasks can be highly repeatable but may approach ceiling performance in healthy samples, limiting their sensitivity to individual differences and experimental effects (Hedge et al., 2018). Conversely, complex multi-component tasks with steep learning curves may show lower stability due to strategy shifts, practice effects, and motivational fluctuations (Duff, 2012; Stanković et al., 2022; Tyburski et al., 2021; Bartels et al., 2010). In practice, tasks should be tuned to avoid floor/ceiling compression and preserve sensitivity to change. Where possible, adaptive titration can help maintain intermediate difficulty (e.g., ~70–80% correct, depending on the staircase rule) across participants (Levitt, 1971).

Timing also matters because tasks performed concurrently with stimulation (online paradigms) may be influenced by ongoing sensory perceptions or distraction, whereas tasks administered after stimulation (offline paradigms) aim to capture aftereffects (Stagg and Nitsche, 2011). As practice effects may accumulate differently across these designs, stability should be evaluated or interpreted separately for online versus offline protocols (Buch et al., 2017; Duff, 2012).

Notably, although motor learning paradigms are extensively studied in tDCS research (Buch et al., 2017), relatively few studies in our mapped sample explicitly quantify across-session sham stability as a primary endpoint. Many trials prioritize active–sham contrasts or single-session effects rather than repeated-sham reproducibility. In addition, rapid acquisition and consolidation-dependent gains complicate separation of sham stability from learning-related change, reinforcing the need for familiarization sessions and explicit modeling when sham stability is a target inference.

Accordingly, behavioral paradigms should demonstrate adequate intrinsic repeatability and well-characterized practice trajectories in pilot testing before deployment in sham-controlled tDCS trials, because otherwise variability under sham cannot be readily separated from measurement noise or residual procedural influences.

### 5.4 Limitations and challenges in sham stability assessment

Sham stability can be affected by confounds that inflate variability, mask stability, or create spurious patterns. These include practice effects, individual/state fluctuations, protocol-related factors, and measurement limitations.

Practice effects are common: early exposures may yield performance improvements that can confound the interpretation of stimulation effects (Hedge et al., 2018). For example, n-back tasks can show meaningful improvements across early sessions without intervention, complicating crossover interpretation when conditions fall within the same learning window (Hedge et al., 2018). Counterbalancing mitigates order effects but assumes symmetric carryover, which may not hold in the presence of nonlinear learning or practice effects (Senn, 2002). Familiarization sessions and mixed-effects models can be applied to partition practice-related variance from stimulation effects (Bates et al., 2015).

Individual variability can also arise from stable traits and fluctuating states (e.g., motivation, alertness, expectancy). Sham-specific contributors include sensory distraction and expectancy-driven changes in effort or performance (Haikalis et al., 2023; Turi et al., 2019). Anatomical differences modulate intracranial electric field distribution and may influence the intensity of perceived sensation, even during brief ramp periods (Opitz et al., 2015). Standardizing session timing and controlling state-dependent factors can reduce within-subject variance, and tracking variables such as sleep, caffeine, or stress, as well as expectancy, may further improve interpretability (Ridding and Ziemann, 2010).

Protocol-related factors can challenge sham inertness. In some devices, low-level background currents may bring the sham closer to a very-low-dose condition than to a strictly zero-current baseline (see Electrophysiological Assessments section) (Nikolin et al., 2018). In addition, group-level connectivity changes have been observed following sham stimulation (Wörsching et al., 2017). Even if smaller than active stimulation, such effects can reduce active–sham contrast and increase unexplained variance (Fonteneau et al., 2019). Cross-over designs also face carryover concerns if active effects outlast washout intervals, potentially contaminating subsequent sham sessions (Nitsche and Paulus, 2001). Device-programmed systems and field-constrained approaches (e.g., HD/multipolar) may help mitigate these risks (Neri et al., 2020; Jog et al., 2025).

Measurement limitations include task impurity and floor/ceiling effects that compress variance and reduce reliability estimates, including ICC (Kane and Conway, 2007; Salkind, 2010). Clinical populations pose additional challenges: performance distributions may differ between groups (e.g., floor effects in patients and ceiling effects in controls), limiting sensitivity and complicating pooled analyses (Hsu et al., 2023). Somatosensory impairment (e.g., altered tactile thresholds) may produce heterogeneous cutaneous perceptions under sham conditions, potentially influencing expectancy and the credibility of control conditions [Contextual extension—outside primary search scope] (Connell et al., 2008).

Finally, high within-subject variability does not necessarily indicate poor measurement quality and it may reflect genuine state fluctuations captured by a sensitive measure instead (Hedge et al., 2018). The goal is to distinguish systematic sources of variability (e.g., practice effects, expectancy, and residual currents that can be modeled) from random noise. Because mean-level change and rank-order stability are distinct properties, both should be reported, with mean trajectories presented alongside consistency metrics (Shrout and Lane, 2012).

## 6 Neurophysiological and neuroimaging reliability of sham tDCS

In addition to behavioral indices, establishing the validity of sham protocols requires careful consideration of neurophysiological and neuroimaging measures, which provide complementary but methodologically distinct readouts of brain activity. Electroencephalography (EEG), functional magnetic resonance imaging (fMRI), and functional near-infrared spectroscopy (fNIRS) index neural activity through distinct biophysical signals. EEG measures voltage fluctuations generated by synchronous postsynaptic potentials in cortical neuronal populations with millisecond temporal resolution, whereas fMRI quantifies task- or state-related changes in blood oxygenation (blood oxygen level–dependent, BOLD, signal), an indirect hemodynamic correlate of neural activity. fNIRS similarly measures changes in concentrations of oxygenated and deoxygenated hemoglobin using near-infrared light, providing a hemodynamic index of cortical activation. Accordingly, the term “activation” in this section refers generically to modality-specific signal changes (electrical or hemodynamic) rather than a unitary physiological process.

### 6.1 Functional MRI and fNIRS studies of sham reproducibility

Neuroimaging provides objective measures of brain activity under sham conditions, thereby contextualizing findings regarding behavioral stability. However, many tDCS neuroimaging studies primarily compare active versus sham conditions rather than explicitly quantifying sham reproducibility across repeated sessions, limiting inferences about baseline neural variance and session-to-session stability (Esmaeilpour et al., 2020).

Although sham is intended to be physiologically inert, greater heterogeneity of individual responses under sham is plausible because measured signals can reflect state-dependent fluctuations (arousal, expectancy, spontaneous network dynamics) rather than consistent neuromodulatory effects. This makes resting-state pre–post comparisons and, ideally, repeat-sham designs particularly informative. However, relatively few studies include RS–sham–RS designs with multiple sham sessions per participant. Furthermore, “no-electrode/no-stimulation” comparator arms remain uncommon in the neuroimaging literature, limiting firm conclusions about the across-day stability of any observed pre- and post-sham differences. Where available, designs incorporating preregistered ROIs, correction for multiple comparisons, and explicit comparators (e.g., sham vs. no-electrode vs. active) provide a stronger basis for determining whether small resting-state changes reflect false positives, nonspecific/placebo influences, or residual current/peripheral mechanisms.

Evidence from multi-session task paradigms suggests that stable task-evoked activation patterns can be observed under control conditions when designs are appropriately standardized. For example, multi-session OLM studies have reported consistent task-related activations in visual association regions across repeated sessions, with moderate-to-good ICC estimates over three sessions (Abdelmotaleb et al., 2025). Such findings support the feasibility of crossover designs when sessions are sufficiently separated (e.g., ≥7 days), while also highlighting that “stable activation” at the group level can coexist with meaningful inter-individual variability and measurement-related variance.

At the same time, some studies report group-level changes following sham that warrant cautious interpretation. Wörsching et al. detected altered resting-state connectivity after sham in frontoparietal networks (Wörsching et al., 2017), highlighting that sham conditions may not be entirely neurophysiologically inert and underscoring the need for careful consideration of analytic flexibility and small effect sizes. More generally, fNIRS signals can exhibit oscillatory and state-dependent variability, reflecting both systemic physiological influences and task or cohort-related factors, underscoring the need to distinguish spontaneous hemodynamic fluctuations from stimulation-related effects (Park et al., 2025; Hoshi, 2007). Across the literature, there remains limited consensus on how best to distinguish subtle sham-related changes from false positives in exploratory, whole-brain analyses.

A related interpretive challenge arises when sham groups show clinical or behavioral improvement. Improvements observed under sham (e.g., in stroke recovery) are often interpreted in light of spontaneous recovery and task practice rather than sham-specific effects (Hsu et al., 2023). However, this attribution is not always directly tested with additional control arms (e.g., no-electrode/no-stimulation comparators), so contributions from peripheral mechanisms or residual device currents cannot be fully excluded in some designs (Boekholdt et al., 2021). Consequently, sham “inertness” should be treated as a design goal supported by evidence, rather than assumed by default.

In summary, fMRI/fNIRS findings under sham can show adequate session-to-session stability for task-based activations in some paradigms (Caumo et al., 2025), but reports of group-level connectivity differences and evidence of hemodynamic variability motivate cautious interpretation and more rigorous multi-arm designs (Wörsching et al., 2017; Park et al., 2025; Antal et al., 2017). Future work would benefit from preregistered ROIs, appropriate correction for multiple comparisons, and explicit comparators (e.g., sham vs. no-electrode vs. active) to strengthen inferences about physiological inertness, particularly given evidence for peripheral contributions to stimulation effects (Boekholdt et al., 2021).

### 6.2 Electrophysiological assessments (EEG, TMS-EEG)

EEG provides millisecond-resolution measures of cortical dynamics that are often expected to remain stable under an inert sham condition. In practice, sham EEG outcomes reflect a combination of measurement reliability, state variability, and the specifics of sham implementation.

Across the literature, EEG measures recorded under sham are often similar to baseline conditions, although some studies report measurable effects even during sham protocols (Vall et al., 2017). However, notable exceptions highlight that some “sham” implementations may not be physiologically null. Nikolin et al. reported that residual background currents (0.016–0.034 mA) present in certain device-programmed sham modes were associated with measurable changes in EEG P3 amplitude relative to unplugged conditions (Nikolin et al., 2018). This finding suggests that, in some implementations, sham may function as a very-low-dose condition rather than a strictly zero-current baseline.

The practical significance of such residual effects likely depends on dose (current magnitude and time-integrated exposure), montage, and analytic sensitivity, and may become detectable in adequately powered designs (Nitsche and Paulus, 2000; Nikolin et al., 2018). In addition, expectancy and contextual factors can modulate neurocognitive and brain-related measures independent of current delivery. For example, placebo or nocebo manipulations have been shown to influence subjective experience and the ability to regulate brain activity, underscoring that psychological effects can affect neural readouts even when stimulation parameters are unchanged (van Elk et al., 2020; Kober et al., 2018). These observations complicate a strict dichotomy between “inert control” and “active comparator” in electrophysiological studies.

Synthesis of the mapped evidence suggests three practical reference points for interpreting sham EEG: (i) a true no-current condition (e.g., unplugged electrodes), (ii) device-implemented sham modes that may include residual monitoring currents, and (iii) expectancy-related modulation that can occur even with identical current delivery. Interpreting EEG findings requires treating these as distinct comparators and being explicit about which sham mode was implemented.

Residual monitoring currents could reduce the active–sham contrast in paradigms where true effects are small, thereby lowering effective signal-to-noise. Where feasible, device-locked systems that support zero-current sham modes can improve interpretability and documentation of protocol fidelity (Kold and Graven-Nielsen, 2023).

### 6.3 Addressing variability in physiological measures

Neurophysiological variability under sham can be conceptualized as arising from multiple sources, including biological factors such as within-subject state fluctuations in arousal, neurotransmitter tone, and cortical excitability (Ridding and Ziemann, 2010), as well as technical (sensor placement, signal-to-noise differences, preprocessing choices) and analytical factors (thresholding, multiple comparison correction, modeling assumptions). Distinguishing these sources is important because they require different mitigation strategies.

Biological variability can be reduced through standardization, including consistent session timing and consideration of state-related factors such as sleep, caffeine, medication, and arousal where feasible (Soleimani et al., 2023). Some state variability is irreducible but can be modeled using random effects rather than treated as noise.

Technical variability can be addressed through standardized procedures and reproducible preprocessing pipelines (e.g., automated, standardized EEG workflows) (Bigdely-Shamlo et al., 2015). Reporting practices, including electrode localization documentation, impedance criteria, and version-controlled analysis code, can further reduce analytical degrees of freedom.

Analytical variability is addressed through transparency and preregistration: preregister analysis plans where possible, adjust for multiple comparisons (e.g., family-wise error rate or false discovery rate), and clearly distinguish confirmatory from exploratory analyses (Ioannidis et al., 2014). Exploratory whole-brain analyses are valuable for hypothesis generation but should be explicitly designated and replicated.

Finally, integrating behavioral and neurophysiological endpoints within the same participants can improve interpretability by enabling mechanistic triangulation. For example, behavioral stability under sham alongside measurable neural variability may clarify whether neural changes are epiphenomenal or behaviorally silent. Although such multimodal designs remain relatively uncommon in the mapped literature, they provide one of the strongest frameworks for testing sham inertness at multiple levels of analysis.

## 7 Blinding efficacy and sensory perception in sham tDCS

Sensory matching between active and sham depends on the ability to replicate peripheral sensations at the electrode sites. This typically includes tingling and itching arising from cutaneous afferent activation during current ramps (Boekholdt et al., 2021). However, sensory profiles can diverge across a session, creating perceptual cues that may compromise blinding in some implementations.

Temporal dynamics of sensation. Conventional FSF protocols generate prominent sensations during ramp-up (e.g., the first 30 s) that often diminish with habituation (Gandiga et al., 2006), whereas active stimulation may be associated with ongoing low-level sensations during sustained current delivery. This temporal mismatch may become more salient over the course of a session (see Section 10 for time-resolved assessment) (Turner et al., 2021). Protocol variants, such as bimodal ramps (initial plus late-session ramp), may improve temporal matching but may still not fully reproduce continuous low-level sensations during active stimulation and may shift sensory salience toward the second ramp (Greinacher et al., 2019; Bjekić et al., 2024). This pattern is consistent with recent data showing temporal variation in perceived unpleasantness when sham includes a late ramp (Bjekić et al., 2024).

Current intensity effects. Higher intensities (e.g., ≥2 mA) can increase sensory divergence between active and sham and may increase discrimination accuracy in some designs (Wallace et al., 2016; Caumo et al., 2025). In HD-tDCS, intensity-dependent variation in perceived sensation has also been reported, potentially reflecting peripheral mechanisms (e.g., skin irritation, erythema) that complicate dose selection and sensory equivalence (Borckardt et al., 2012).

Assessment timing and interpretation. Apparent “more sensation” does not necessarily indicate active stimulation. For example, El Jamal et al. reported higher rates of burning, tingling, and itching after sham than after active HD-tDCS in a large-session dataset (El Jamal et al., 2023). One plausible explanation is that a late-session ramp in sham may have occurred closer to the time of sensation assessment, whereas active stimulation may have allowed greater habituation prior to assessment. This highlights that sensory reporting depends strongly on the timing of the most recent salient sensory event relative to the measurement.

Montage effects. Sensation intensity can vary by montage. Some conventional bifocal configurations may produce stronger localized sensations than multifocal HD approaches at comparable total current. This is consistent with the observation that current density is higher at fewer stimulation sites (Neri et al., 2020). This creates a practical trade-off. While conventional montages may be simpler to implement, they can be harder to blind, whereas HD montages may improve sensory control but require specialized equipment. ActiSham, which uses tangential scalp current paths to generate sensations while minimizing cortical penetration, has been reported to achieve sensory equivalence to multifocal stimulation without detectable changes in corticospinal excitability, offering a proof of principle for physics-constrained sham design (Neri et al., 2020).

As an alternative strategy, splitting the current across adjacent HD electrodes (e.g., dividing 2 mA into two 1 mA channels) may help equalize sensations between active conditions without requiring a separate sham condition. This approach can simplify implementation but may limit placebo isolation (Richardson et al., 2014).

Sham versus active control. Sham primarily controls expectancy, context, and cutaneous sensations while attempting to avoid physiological neuromodulation. This makes it well-suited for isolating effects attributable to current delivery beyond nonspecific factors. In contrast, an “active control” (e.g., an alternative montage, a different site, or a different waveform) may better match the sensory experience but can introduce its own physiological effects, complicating causal interpretation. Where feasible, factorial designs (active vs. sham × site/waveform) can help separate sensory/context effects from neurophysiological specificity.

Synthesis. Sensory matching depends on temporal profile fidelity (bimodal or sustained strategies may outperform single ramps), stimulation intensity, montage configuration, and assessment timing (Gandiga et al., 2006; Neri et al., 2020; Turner et al., 2021; Greinacher et al., 2019; Wallace et al., 2016; El Jamal et al., 2023; Duff, 2012; Caumo et al., 2025). No single sham protocol optimizes all dimensions simultaneously. Therefore, protocol choice should reflect study priorities and the planned blinding assessment strategy.

A practical implementation template for time-resolved blinding assessment is provided in Box 1 (Section 10).

## 8 Methods for assessing blinding integrity

Blinding assessment should occur at multiple time points, with prespecified criteria to detect failure when it occurs, rather than relying solely on retrospective end-of-study estimates, which are subject to recall and response biases (Turner et al., 2021; Greinacher et al., 2019). Time-resolved forced-choice probes (active vs. sham) with confidence ratings can be administered at predefined time points during stimulation (e.g., immediately after the initial ramp, mid-session, late-session) and analyzed using mixed-effects approaches (Turner et al., 2021; Greinacher et al., 2019). For a conventional fade-in/short-stimulation/fade-out (FSF) sham, forced-choice questions should be phrased at the session level and should not suggest that stimulation may switch on/off mid-session unless that is genuinely possible in the protocol.

BOX 1 **Time-resolved blinding assessment protocol (implementation template).**

Objective: Detect unblinding during stimulation rather than relying solely on retrospective end-of-study guesses (Turner et al., 2021; Greinacher et al., 2019).

Timepoints (example): T0 (~1 min), T1 (~10 min), T2 (~18–20 min).

Measures: forced-choice allocation guess, confidence rating, and sensory VAS ratings.

Analysis (pre-specified): mixed-effects models for guess accuracy over time.

Where feasible, equivalence-style criteria can be pre-registered to define what constitutes “acceptable” blinding performance, rather than interpreting null differences *post hoc*.

Equivalence testing (TOST) and margins. Equivalence testing using the two one-sided tests (TOST) framework evaluates whether an observed difference is smaller than a prespecified equivalence margin (±*Δ*), where Δ represents the smallest difference considered practically meaningful (i.e., the smallest effect size of interest). Rather than testing whether a difference is exactly zero, TOST tests whether the effect lies entirely within (−Δ, +Δ). If both one-sided tests are significant (equivalently, if the confidence interval falls fully within the margins), results support practical equivalence within the chosen Δ. Equivalence margins should be justified *a priori* (e.g., a prespecified VAS difference for sensations or a standardized effect size threshold) and reported transparently.

Repeated sensory ratings during stimulation: online vs. offline implementation. Repeated ratings of itch, tingling, warmth, and pain can be collected without materially disrupting task performance if probes are brief and scheduled. In online paradigms (task during stimulation), sensory ratings should be sampled during pre-planned micro-pauses or between task blocks (e.g., a 3–5 s VAS prompt), rather than during time-critical trials, to minimize attentional capture and performance interference. In offline paradigms (task after stimulation), sensory ratings can be sampled at standardized stimulation timepoints (e.g., immediately after ramp-up, mid-stimulation, and pre-ramp-down) without competing with task demands. In both cases, probe timing should be fixed across conditions, kept as brief as possible, and analyzed with models that account for time (and protocol phase) to avoid misattributing time-dependent sensory changes to stimulation effects.

*Parallel sensory assessment*. Sensory ratings (VAS for itch, tingling, warmth, pain) collected at matching time points provide complementary evidence regarding perceptual cues. Where the goal is to demonstrate similarity (rather than merely “no difference”), sensory equivalence can be evaluated using prespecified equivalence margins (e.g., via TOST), shifting interpretation from null-hypothesis testing to evidence of practical similarity.

*Design-specific considerations*. Within-subject crossover designs are vulnerable to comparison effects across sessions (O’connell et al., 2012). Mitigation strategies include longer washout periods, session-specific assessment of expectations, and careful consent and briefing procedures. Any use of deception requires appropriate ethical justification (including alignment with ethical and legal guidance for low-intensity tES) and debriefing (Antal et al., 2017).

*Experimenter blinding*. Experimenter blinding is often under-assessed but can influence outcomes. Where feasible, triple-blind procedures with separate personnel for electrode application, device operation, and outcome assessment may help reduce bias (Arroyo-Fernández et al., 2021; Loreti et al., 2023). Operator guesses and confidence ratings can be collected and reported to evaluate the integrity of experimenter blinding, recognizing that visible cues (e.g., erythema) may contribute to inadvertent unmasking (Ezquerro et al., 2017; El Jamal et al., 2023). The added value of device-locking and triple-blind procedures is greatest for subjective or clinician-rated outcomes (e.g., symptoms, mood scales), whereas for fully computerized performance endpoints it primarily mitigates inadvertent cueing and protocol deviations rather than outcome scoring bias.

*Statistical reporting*. Transparent reporting may include Bang’s Blinding Index and James’ Blinding Index with confidence intervals and raw contingency tables (Dedoncker et al., 2016; Jamil et al., 2017). Simple chi-square comparisons can miss asymmetric unblinding patterns (e.g., correct identification of active but not sham) that require a different interpretation (Dedoncker et al., 2016; Jamil et al., 2017).

We recommend preregistering blinding timepoints, metrics, analysis plans, and decision rules, because without pre-specification interpretation can become subjective and prone to confirmation bias.

### 8.1 Challenges and advances in achieving effective blinding

Conventional FSF sham may be vulnerable to blinding failure, particularly in multi-session designs and among participants with prior exposure to tDCS-like sensations (Sheffield et al., 2022; O’connell et al., 2012). Several advances aim to improve blinding integrity.

#### 8.1.1 Device-locked programmable shams

Device-locked and code-based allocation may reduce operator influence and improve auditability (Jog et al., 2025). Modern systems may log impedance, delivered current, and timestamps, enabling *post hoc* verification of protocol adherence and supporting multi-session and multi-region stimulation designs (Kold and Graven-Nielsen, 2023; Jog et al., 2025).

#### 8.1.2 HD/multipolar and field-constrained approaches

HD-tDCS and multipolar configurations can shape current paths to better constrain cortical fields and may help control sensory profiles (Richardson et al., 2014). ActiSham has been described as minimizing intracranial fields through optimized geometry, while maintaining scalp sensations, with reports suggesting limited physiological effects on corticospinal excitability (Neri et al., 2020). These approaches highlight the potential for physics-informed sham design.

#### 8.1.3 Expectancy management

Expectancy and contextual factors contribute to blinding outcomes and may be moderated through neutral consent language, standardized staff scripts, and structured sensory rating procedures (Stanković et al., 2022). Such strategies do not eliminate expectancy effects but can reduce inadvertent cueing.

#### 8.1.4 Unresolved challenges and trade-offs

Prior tDCS exposure may increase discrimination ability across sessions (O’connell et al., 2012). In addition, residual monitoring currents in some commercial sham modes raise the possibility that better sensory matching may be achieved with small physiological non-inertness in some implementations (Nikolin et al., 2018). These trade-offs motivate transparent reporting of sham parameters, sensory cues, and (where feasible) physiological checks.

#### 8.1.5 Path forward

Next-generation shams should aim to jointly optimize sensory equivalence and field minimization using computational modeling and empirical validation, rather than assuming that matched sensations imply inertness. Where feasible, preregistered behavioral blinding criteria and physiological criteria (e.g., conservative engineering ceilings and/or neutral neurophysiological markers) can strengthen inference in sham validation studies.

## 9 Inter-individual and contextual factors influencing sham responses

In addition to inter-study variability, sham responses can vary across individuals and sessions due to anatomical features, state-dependent factors, and experimental context (Vergallito et al., 2022; Polanía et al., 2018).

### 9.1 Biological variability among participants

Sham response variability reflects both stable anatomical traits and fluctuating physiological states, which can influence sensory perception and, depending on the montage and dose, intracranial field exposure during sham ramps.

#### 9.1.1 Anatomical factors

Individual differences in head anatomy (e.g., scalp-to-cortex distance, skull thickness, and tissue conductivity) can influence both perceived sensations and the magnitude and distribution of intracranial electric fields generated by a given montage (Opitz et al., 2015; López-Alonso et al., 2015; Thair et al., 2017). Such variation can alter intracranial exposure even under nominally identical sham parameters and may increase the risk of unblinding through differences in perceived sensations (Opitz et al., 2015). Modeling studies indicate that anatomical variability can meaningfully affect predicted cortical field magnitudes under identical stimulation settings (Laakso et al., 2015), suggesting that the sham “dose” can vary across individuals even when device settings are fixed.

#### 9.1.2 Actionable solutions

Individualized head models derived from structural MRI can be used to estimate electric fields and characterize inter-individual variability (Datta et al., 2009). When MRI is not feasible, proxy measures, such as scalp-to-cortex distance at target sites, have been proposed as pragmatic alternatives (Lu et al., 2019). Without such measures, anatomical variability remains an unmeasured confound.

#### 9.1.3 Age and clinical population effects

Sensation intensity and tolerance may differ by age and clinical status. Some studies report lower sensation intensity in older adults (Wallace et al., 2016), potentially reflecting age-related changes in cutaneous afferent function. Evidence from clinical cohorts suggests that blinded performance can be maintained under adequately controlled protocols, although results can vary by design and population (Loo et al., 2018; Gorsler et al., 2021).

#### 9.1.4 Circadian timing (time-of-day)

Circadian factors can influence arousal, pain/sensory perception, autonomic tone, and cognitive performance, prompting session scheduling at consistent times when feasible and recording the time of day to enable adjustment or sensitivity analyses.

#### 9.1.5 Other factors

Findings regarding sex, gender-related effects, and hormonal influences remain inconsistent across studies (Vergallito et al., 2022; Dedoncker et al., 2016; Jamil et al., 2017). In the absence of robust, reproducible patterns, routine tracking may be best reserved for targeted mechanistic studies rather than applied universally. Other attributes, such as hair properties and skin features, can influence comfort and visible erythema, but their relationship to blinding success remains unclear across controlled settings (El Jamal et al., 2023). These factors may become more salient in home-based or repeated-use protocols but are secondary in many laboratory designs.

In summary, where feasible, measuring or modeling anatomical features (e.g., scalp-to-cortex distance) and accounting for age may improve design and interpretation. Other sources of variability may be acknowledged as potential contributors without imposing excessive measurement burden that could reduce feasibility and compliance.

### 9.2 Contextual and experimental variables

Beyond stable biological traits, session-level context and procedural choices may introduce state-dependent variance that can often be controlled experimentally.

#### 9.2.1 Expectancy effects

Participants’ beliefs about receiving active versus sham stimulation can influence subjective experience and objective neural measures. Placebo or nocebo manipulations have been associated with changes in brain-related and neurocognitive outcomes under identical stimulation parameters, indicating that psychological context can have measurable neurophysiological correlates (van Elk et al., 2020; Kober et al., 2018). At the same time, expectancy-related differences do not uniformly translate to differences in objective task performance across all paradigms. Some studies report similar behavioral outcomes regardless of whether correct allocation guesses are made (Stanković et al., 2022). Subjective outcomes (e.g., pain, mood, perceived effort) may be more susceptible to expectancy-driven effects (Turi et al., 2019).

Where appropriate, expectancy can be assessed explicitly (e.g., belief about allocation and confidence) and included as a covariate. Standardized, neutral instructions may reduce inadvertent cueing, and concurrent tasks may reduce sustained attention to electrode sensations (Greinacher et al., 2019).

#### 9.2.2 Procedural standardization

Technical variance can arise from electrode placement precision, impedance consistency, and preparation procedures (Woods et al., 2016). Photographic documentation, neuronavigation (when available), and impedance logging can support quality control and facilitate detection of procedural outliers. Studies that implement rigorous standardization may report reduced outcome variance compared with less controlled protocols (Fonteneau et al., 2019).

#### 9.2.3 Task and timing interactions

Attention to stimulation-related sensations may vary over the course of a task (Greinacher et al., 2019). More demanding tasks may reduce awareness of task-irrelevant sensory cues relative to passive conditions (Chen et al., 2017). The timing of stimulation relative to tasks (online vs. offline) can also influence the balance between stimulation effects during task performance and aftereffect measurement (Stagg and Nitsche, 2011). Session timing can contribute to state-related variability in repeated-measures designs (Ly et al., 2016).

In summary, consistent scheduling and control of state-related factors can reduce avoidable variance and improve reproducibility (Ridding and Ziemann, 2010).

### 9.3 Implications for sham protocol design and analysis

These sources of variability motivate a tiered strategy: essential controls applicable to most studies, enhanced controls for well-resourced trials, and exploratory measures for mechanistic work.

Tier 1: Essential controls.

- Demographic stratification: Stratify or balance by age and clinical status where relevant.
- Procedural standardization: Standardize electrode placement and preparation procedures and document impedance and setup parameters to support quality control (Woods et al., 2016).
- Expectancy assessment: Brief session-level expectancy measures (belief, confidence, expected benefit) can support covariate adjustment where appropriate.
- State monitoring (minimal): record sleep (previous night), caffeine/nicotine intake, and any medication changes prior to each session.
- Session timing: Schedule repeated sessions at similar times of day to reduce circadian-related variability.

Tier 2: Enhanced controls.

- Anatomical characterization: Use structural MRI-based modeling or proxy measures (e.g., scalp-to-cortex distance) to support field estimation (Datta et al., 2009; Lu et al., 2019).
- State monitoring (expanded): collect richer state measures (e.g., standardized sleepiness/stress scales, actigraphy where feasible, or additional physiological measures) and model these as time-varying covariates.
- Blinding robustness: Where feasible, implement triple-blind procedures to reduce experimenter influences (Arroyo-Fernández et al., 2021).

#### 9.3.1 Analytical strategies for heterogeneity

When variability sources are measured, mixed-effects modeling can accommodate inter-individual differences while estimating population-level effects (Bates et al., 2015). Covariate adjustment (e.g., age, anatomy proxies, expectancy) can improve precision. For residual heterogeneity, sensitivity analyses can be pre-registered (e.g., robust estimators, stratified analyses) to reduce *post hoc* flexibility.

#### 9.3.2 Critical principle

Avoid post hoc exclusion based on “anomalous” sham responses without pre-specified criteria. Instead, pre-register decision rules and report analyses with and without outlier-handling approaches transparently.

#### 9.3.3 Reporting transparency

Report distributions and individual trajectories where feasible, not only means, to clarify whether null sham effects reflect true inertness or cancelation across heterogeneous responses (Shrout and Lane, 2012).

## 10 Cross-modal context: tACS blinding considerations

As noted in Methods (Section 4.2), tACS is discussed here as a contextual extension beyond the primary tDCS-focused search scope, to illustrate sham/blinding principles that may generalize.

### 10.1 Physiological and perceptual differences between tDCS and tACS

tACS delivers sinusoidal alternating current at specified frequencies rather than constant polarization, creating distinct challenges for sensory masking and blinding (Soleimani et al., 2023; Antal and Paulus, 2013). Two perceptual differences are particularly relevant to sham design: frequency-dependent cutaneous sensations and the potential for phosphene induction.

#### 10.1.1 Frequency-dependent sensory profiles

Unlike tDCS, where perceived sensation often scales more directly with amplitude, tACS perception can vary with stimulation frequency as well as amplitude (Antal and Paulus, 2013). This creates frequency-specific masking challenges, where a sham designed for one frequency may not transfer to another without adjustment. Comparative evidence suggests that perceived sensation intensity is a major determinant of unblinding across transcranial electrical stimulation modalities (Sheffield et al., 2022).

This consideration is further complicated by the heterogeneous recruitment and adaptation profiles of peripheral sensory fibers, which may differentially respond to waveform and frequency parameters. Consequently, sham designs must account not only for perceptual plausibility but also for the neurophysiological pathways through which afferent signaling may persist. Cutaneous percepts during transcranial electrical stimulation are mediated by heterogeneous populations of sensory fibers with distinct activation thresholds and adaptation kinetics. Large-diameter Aβ mechanoreceptive afferents typically exhibit rapid adaptation, whereas thinly myelinated Aδ and unmyelinated C fibers, implicated in tingling, burning, or itching sensations, display slower or incomplete adaptation profiles (Basbaum et al., 2009). Because alternating waveforms may differentially recruit peripheral afferents as a function of frequency and temporal dynamics, the qualitative and temporal evolution of sensation in tACS can diverge from that observed under constant-current tDCS. Empirical characterization of tDCS-induced scalp sensations demonstrates that perception of intensity and quality evolves over time, consistent with differences in underlying peripheral mechanisms (Kessler et al., 2012). These peripheral dynamics have direct implications for sham design since their matched initial perceptual intensity may not ensure equivalence in adaptation or sustained afferent signaling across modalities.

#### 10.1.2 Phosphenes (retinal stimulation)

Depending on montage and parameters, tACS can induce visual precepts (phosphenes), particularly when stimulating near the occipital regions and within commonly used frequency bands (Matsumoto and Ugawa, 2017; Kanai et al., 2008). These precepts introduce an additional cue beyond scalp sensations and therefore impose constraints on sham design (e.g., montage selection and parameter choices intended to minimize visually salient cues).

#### 10.1.3 Mechanistic uncertainty and sham implications

Whether tACS effects arise predominantly from direct cortical entrainment versus peripheral pathways remains debated (Asamoah et al., 2019). If peripheral mechanisms contribute meaningfully, then a sham that merely matches scalp sensation could, in principle, produce physiological effects through non-transcranial routes. This parallel concerns tDCS and residual currents, as well as peripheral contributions, and reinforces the broader point that perceptual plausibility does not automatically imply physiological inertness.

In conclusion, the key insights transferable across modalities suggest that matching sensory experience is not sufficient to guarantee physiological neutrality. Where peripheral activation is likely to contribute, sham approaches may need to constrain current paths and field exposure (e.g., geometry-driven shunting and lower effective intracranial fields) rather than focusing only on temporal sensory matching.

### 10.2 Sham methodologies in tACS research

tACS sham protocols parallel common tDCS approaches and are characterized by brief ramps, low-intensity matching, or bimodal delivery. However, they require adaptations that are mindful of stimulation frequency (Vossen et al., 2015). A key design principle emerging from cross-modal work is the use of field-shaping approaches intended to maintain sensory plausibility while minimizing intracranial exposure. ActiSham exemplifies this logic by using controlled, geometry-informed current delivery to produce plausible sensations while minimizing physiological effects in validation paradigms (Neri et al., 2020). Although developed in multifocal tDCS contexts, the underlying principles (field shaping and controlled sensory matching) are also transferable to AC waveforms.

It has been suggested that ActiSham may achieve sensory equivalence to active multifocal stimulation while producing limited changes in corticospinal excitability in specific validation settings (Neri et al., 2020). More broadly, these findings support the feasibility of physics-informed sham design that targets both perceptual plausibility and physiological neutrality, rather than relying solely on ramp-based masking.

A tACS-specific challenge is phosphene control. Some studies have used topical anesthetics to reduce peripheral activation in mechanistic experiments, but this approach may be impractical for clinical protocols and can introduce new blinding cues (Asamoah et al., 2019). Alternative strategies include montage and parameter choices intended to reduce visually salient features, although such choices can constrain mechanistic hypotheses regarding frequency-specific entrainment.

Comparative evidence indicates that blinding in tACS can be challenging when sensations are stronger at comparable intensities. Additionally, sensation intensity rather than waveform per se remains an important predictor of unblinding across modalities (Sheffield et al., 2022). These observations support the relevance of shared optimization strategies (e.g., montage choice, intensity management, and time-resolved assessment) while preserving the distinct constraints introduced by phosphenes and frequency dependence.

## 11 Emerging technologies and a technical roadmap for better sham tDCS

Current sham tDCS protocols have often evolved pragmatically. Brief ramps were chosen for convenience, sensory similarity was assessed primarily via self-report, and physiological inertness was frequently inferred rather than directly verified. A next-generation approach is to invert this logic: start with explicit design constraints (e.g., conservative intracranial field ceilings and sensory-equivalence targets), use computational modeling to meet them, and validate their effectiveness with objective readouts. This section outlines a technical roadmap grounded in physics and engineering, and supported by transparent reporting practices, that can be implemented using existing or near-term tools (see Box 2 for a concise, operational framework).

BOX 2 **Evidence-informed design principles for specification-grade sham tDCS (proposed operational framework).**

*This framework is intended as a practical checklist derived from recurring methodological issues and solutions mapped in the reviewed literature; it is not a prescriptive standard.*

1. Sensory equivalence with pre-specified margins. Aim to match the time course and intensity of cutaneous sensations between active and sham conditions. Where used, bimodal ramps and matched electrode preparation, as well as impedance procedures, can reduce differential cues (Arroyo-Fernández et al., 2021; El Jamal et al., 2023). Pre-register sensory equivalence margins and evaluate using equivalence-oriented approaches rather than relying on non-significant differences alone.
2. Constrain intracranial electric fields. Where feasible, use modeling tools (e.g., SimNIBS, ROAST) to set and document a conservative engineering ceiling for sham intracranial fields and to design current paths that preferentially minimize cortical penetration (Opitz et al., 2015). Document modeling assumptions (e.g., conductivity parameters and mesh choices) for reproducibility.
3. Prefer field-shaped multipolar shunting when available. HD/multipolar montages can support extracranial current steering and may better support joint optimization of sensory plausibility and field minimization than standard bipolar approaches in some contexts (Neri et al., 2020; Richardson et al., 2014).
4. Device-locked randomization with audit trails. Use device-level allocation concealment and automated protocol delivery where available (Jog et al., 2025). Retain device logs capturing impedance, delivered current, and timestamps to support *post hoc* verification and multi-region stimulation reproducibility.
5. Time-resolved blinding assessment. Embed brief, pre-specified blinding probes during the session (e.g., early, mid-session, late) rather than relying solely on end-of-study guesses (Turner et al., 2021; Greinacher et al., 2019). Where appropriate, define and preregister criteria for acceptable blinding performance.
6. Sensory measurement at multiple timepoints. Collect sensory ratings at matched timepoints to characterize temporal profiles and identify mid-session divergence, a common failure mode for ramp-only approaches (Turner et al., 2021).
7. Session-level quality assurance. Record and archive key procedural parameters (electrode placement, impedance trajectories, contact medium, adverse events) using structured forms to support quality control and sensitivity analyses (Woods et al., 2016).
8. Transparent reporting for reproducibility. Report ramp shapes, durations, current densities, electrode materials, sizes, contact medium, and deviations from planned protocols. Where feasible, share modeling files and analysis code to facilitate replication and cross-study aggregation.

### 11.1 From assumption to specification: physics-constrained design

We argue that the foundational shift necessitates treating sham inertness as a measurable specification rather than an implicit assumption. A physics-constrained workflow can include: (1) pre-registering an intracranial E-field ceiling expressed as a conservative engineering target (e.g., peak field below a specified value at the intended cortical region); (2) using individualized or representative head models to design electrode configurations that meet this ceiling while achieving an acceptable sensory profile; (3) verifying device outputs and current delivery characteristics against the intended protocol; and (4) confirming, where feasible using sensitive neurophysiological readouts, that sham effects remain below the detection limits relevant to the study endpoints (Opitz et al., 2015).

The specific numerical value selected for an E-field ceiling should be justified and treated as a design target rather than a biological threshold. Candidate values are informed by computational modeling studies demonstrating the spatial variability of induced electric fields and by empirical work linking field magnitude to measurable neurophysiological effects, while acknowledging that consensus remains incomplete and outcome sensitivity varies across paradigms (Bikson et al., 2016; Opitz et al., 2015). The key is explicit specification and verification, not any single universal cutoff. Accessible tools (e.g., SimNIBS, ROAST) support such modeling in standard research settings, and emerging device ecosystems are beginning to incorporate field-estimation workflows (Huang et al., 2019). In many cases, the primary barrier is the culture of implementation and reporting norms rather than feasibility.

### 11.2 Multipolar current steering: sensation without penetration

HD-tDCS systems with 4 × 1 or higher-density arrays enable field-shaping strategies that preferentially distribute current across the scalp while constraining intracranial field exposure (Datta et al., 2009). This leverages the practical observation that sensory experience can be elicited by peripheral activation under conditions in which intracranial fields are minimized, thereby creating a design window for sham optimization (Boekholdt et al., 2021).

ActiSham illustrates the principle that continuous, low-intensity delivery via an optimized multipolar geometry elicits sensations indistinguishable from active 4 × 1 stimulation, yet has minimal effects on corticospinal excitability in validation studies (Neri et al., 2020). Importantly, such approaches are compatible with computational optimization. They define a cost function that increases scalp-level current density (sensory plausibility) while minimizing intracranial field energy (penetration). Conceptually, this optimization framework can be generalized to multiple targets and montages, although broader validation across devices and paradigms remains a priority.

Implementation typically requires multi-channel systems and flexible current control. Devices such as Soterix MxN and Neuroelectrics Starstim support this capability, though many studies use these systems primarily for focal active stimulation rather than explicitly optimized sham configurations. Wider uptake may be supported by shared modeling pipelines and pre-computed montage libraries for common targets (e.g., M1, DLPFC), as well as clearer reporting standards.

### 11.3 Temporal profiling: bimodal ramps and waveform shaping

Single-onset ramps can produce salient initial sensory cues but diverge from the temporal profile of active stimulation, thereby contributing to time-dependent unblinding detected by time-resolved probes (Turner et al., 2021; Greinacher et al., 2019). Bimodal ramps (brief stimulation at the beginning and end of a session) are one strategy intended to better approximate the temporal envelope of sensation, although their effectiveness likely depends on intensity, montage, participant characteristics, and the timing of sensory assessment (El Jamal et al., 2023).

Waveform shaping offers further potential refinement. Variations in ramp profiles or programmed modulation may help mimic the variability of perceived sensation without materially increasing time-integrated current, depending on implementation (Woods et al., 2016). Such approaches typically require programmable devices beyond fixed ramp parameters and benefit from preregistering waveform specifications to distinguish planned design from *post hoc* adjustments. The key trade-off is that additional ramp phases introduce additional current delivery. In such a case, modeling and validation should verify that improved sensory matching does not meaningfully compromise field-constrained inertness.

## 12 Future directions: empirically testable propositions, study designs, and checklists

This section distills the most consequential evidence gaps, outlines study designs that could resolve them, and provides a pragmatic implementation checklist for investigators planning sham-controlled trials.

### 12.1 Synthesis and empirically testable agenda for next-generation sham validation

The mapped literature indicates that conventional sham protocols can exhibit time-dependent sensory divergence, variable blinding performance across designs, and, in some implementations, signals that are difficult to distinguish from residual physiological or analytic artifacts. At the same time, the literature also points to technically feasible pathways to improvement (e.g., device-locked delivery, time-resolved blinding assessment, and field-constrained/field-shaped approaches). The key implication is that sham selection and validation should be treated as an empirical design problem rather than a default control assumption: investigators should specify which property of sham is most critical for their inference (sensory plausibility, physiological inertness, auditability, or multi-session stability) and choose an approach aligned to that priority.

The first evidence gap concerns how to assess blinding. Existing support for time-resolved probes is concentrated in relatively few studies, and it remains uncertain when within-session probing provides decisive information beyond end-of-study guesses across populations, intensities, and montages (Turner et al., 2021; Greinacher et al., 2019). This motivates prospective designs that directly compare time-resolved versus retrospective blinding metrics within the same protocol, with prespecified criteria for acceptable blinding and a plan for how blinding outcomes will be handled analytically (e.g., sensitivity analyses, predefined per-protocol sets).

A second gap is the shortage of head-to-head comparisons of sham protocol families under controlled conditions. Without direct comparisons that hold population, task, and session structure constant, it is difficult to justify “ranking” shams or generalizing from protocol-specific demonstrations. A practical agenda is therefore to conduct within-subject testing of multiple sham variants (e.g., FSF, bimodal ramps, and field-shaped HD/multipolar shams), with matched tasks, standardized preparation, and outcomes that capture both sensory time-courses and time-resolved blinding performance.

A third gap concerns the empirical validation of modeling-based intracranial field targets. Although conservative ceilings have been proposed, the field does not yet converge on a single magnitude that can be treated as negligible across behavioral and neurophysiological endpoints. Dose–response work is needed in which sham conditions are explicitly defined by model-estimated exposure bands (from conservative to higher exposure), ideally benchmarked against a no-current comparator when feasible, to identify the exposure range at which measurable effects begin to emerge for the outcomes under study.

Finally, sham validity over extended treatment schedules remains incompletely characterized. Most reliability and blinding evidence derive from relatively short multi-session designs, whereas clinical schedules can involve 10–20 sessions. Learning effects, cumulative expectancy, and skin-related changes may alter sensory cueing and discrimination across time, suggesting that extended protocols should incorporate periodic blinding probes, structured adverse-event tracking, and (where feasible) repeated physiological checks in a subset. Collectively, these gaps define a falsifiable and decision-relevant agenda: (i) quantify when time-resolved assessment changes inference, (ii) compare sham families directly, (iii) validate modeling-based exposure ceilings against outcomes, and (iv) test sham stability across extended schedules.

### 12.2 Empirically testable propositions emerging from the mapped evidence

The mapped evidence supports several falsifiable, decision-relevant propositions that can be evaluated in future sham validation studies:

1. Time-resolved blinding probes will detect within-session divergence that end-of-study guesses miss, particularly in multi-session designs and in protocols where sensory profiles diverge during sustained stimulation phases (Turner et al., 2021; Greinacher et al., 2019).
2. Temporal sensory-matching variants (e.g., bimodal strategies, when appropriate) will improve sensory equivalence relative to single-ramp FSF approaches, but may shift sensory salience toward specific protocol transitions, necessitating time-resolved assessment.
3. Field-constrained or field-shaped sham strategies that explicitly target low intracranial exposure will reduce the likelihood of sustained neurophysiological changes beyond transient ramp-related artifacts, improving the interpretability of sham baselines in sensitive measures.
4. Blinding integrity will drift over repeated sessions as participants learn stimulation-associated cues, especially among participants with prior tDCS experience, implying that experience and order effects should be treated as first-order design factors rather than nuisance variance.

These propositions are intentionally framed as testable and decision-relevant, while acknowledging that the current mapped evidence base does not support precise quantitative forecasts.

## Appendix A Implementation checklist/decision guide

Practical decision checklist for selecting and validating sham protocols in tDCS studies.

**Table:**

Domain (trigger)	Minimum implementation	Escalation when sham validity is central
Blinding assessment *(online endpoint/high cue risk)*	End-of-session forced-choice + confidence; document standardized pre-session script.	Time-resolved probes (early/mid/late) + mixed-effects modeling; prespecified “acceptable blinding” criteria (Turner et al., 2021; Greinacher et al., 2019).
Sensory matching *(≥2 mA, long sessions, prior tDCS exposure)*.	Sensory VAS (itch/tingling/warmth/pain) at standardized timepoints	Time-resolved sensory trajectories; consider temporal-matching variants (e.g., bimodal where appropriate) + preregistered margins for sensory similarity
Physiological inertness *(mechanistic physiology/imaging claims)*	Report sham waveform/ramp parameters and device mode in full	Preregistered ROIs/physiological checks; add neutral comparator (no-electrode/no-current) where feasible
Modeling/field constraints *(field minimization is a design goal)*	Report modeling assumptions + estimated fields (active and sham)	Prespecified conservative exposure targets; empirical validation in a subset (dose/exposure bands when feasible)
Technical QA *(multi-session/multichannel/multi-region)*	Log impedance + setup parameters; prespecify acceptable ranges; flag deviations	Device-locked randomization + auditable logs; prespecified protocol-adherence checks
Expectancy/ethics *(risk of cueing or deception)*	Neutral consent language; standardized staff scripts; debriefing plan if deception used	Session-specific expectancy measures; sensitivity analyses stratified by experience/exposure
Transparency/reproducibility *(all studies)*	Report sham parameters with the same detail as active (electrodes, prep, ramps, criteria, deviations)	Share anonymized blinding/sensory-probe data; preregistration where feasible
