# Supplementary material for: Sham tDCS controls: blinding, reliability, and a specification-grade checklist
Source: Front Hum Neurosci. 2026 May 5;20:1742370. doi: 10.3389/fnhum.2026.1742370 (PMC13183852; doi:10.3389/fnhum.2026.1742370)
Supplement: Supplementary file 1 [file Data_Sheet_1.PDF]

Detailed Search Strategy

1. Overview

Literature searches were conducted in PubMed/MEDLINE, Scopus, Web of Science, and PsycINFO from database inception through August 31, 2024. A targeted post-search update was performed in September 2025 (covering September 2024–September 2025) to capture newly indexed methodological advances directly relevant to sham implementation, blinding integrity, physiological inertness, and reliability under sham conditions.

The search strategy was structured around predefined conceptual blocks and combined controlled vocabulary (e.g., MeSH terms in PubMed) with expanded free-text synonyms. Boolean operators (AND/OR) were used to organize and combine related constructs. The search was designed to ensure broad conceptual coverage and methodological representation rather than exhaustive systematic enumeration.

2. Conceptual Block Structure

Search terms were grouped into the following conceptual domains:

Block 1: Stimulation Modality

- “transcranial direct current stimulation”
- tDCS
- “HD-tDCS”
- “high-definition tDCS”

Block 2: Sham / Control Condition

- sham
- “sham stimulation”
- placebo
- “placebo stimulation”
- control
- “active sham”
- “ramp sham”
- masking

Block 3A: Blinding / Sensory Integrity

- blind*
- blinding
- “blinding assessment”
- masking
- expectancy

- guess
- "time-resolved"

Block 3B: Reliability / Reproducibility

- reliability
- "test-retest"
- reproducibility
- repeatability
- stability
- "intraclass correlation"
- ICC

Block 3C: Physiological Inertness / Objective Measures

- inert
- inertness
- physiological
- EEG
- ERP
- "event-related potential"
- fMRI
- "functional magnetic resonance imaging"
- fNIRS
- "near-infrared spectroscopy"
- TMS
- MEP
- "motor evoked potential"

---

3. Boolean Logic Structure

The general Boolean framework used across databases followed this structure:

(Block 1: Stimulation modality)
AND
(Block 2: Sham/control terminology)
AND
(Block 3A OR Block 3B OR Block 3C)

Database-specific syntax and subject headings were adapted according to each platform's indexing architecture.

5. Targeted Post-Search Update (September 2024–September 2025)

A focused update search was conducted in September 2025 using the same Boolean structure, with additional emphasis on emerging methodological terminology, including:

- "device-locked"
- "current steering"
- "multipolar"
- "field-constrained"
- "time-resolved blinding"

The update search was restricted to publications indexed between September 1, 2024 and September 30, 2025. Newly identified records were screened for conceptual relevance to sham methodology, blinding integrity, physiological inertness, and reliability endpoints, and incorporated where they provided substantive methodological insight.

---

6. Citation Tracking

Backward and forward citation tracking was conducted from sentinel methodological papers (13,14,16) and relevant recent reviews (35,36) to identify influential methodological exemplars not captured through keyword-based retrieval.

---

7. Scope Clarification

Consistent with the narrative scoping design of the review, the search strategy was structured to ensure breadth and methodological representation rather than exhaustive systematic capture of all eligible studies. Study inclusion was guided by conceptual relevance to sham implementation, blinding assessment, physiological inertness, and reproducibility domains.
